# Innate Vascular Failure by Application of Neuroleptics, Amphetamine, and Domperidone Rapidly Induced Severe Occlusion/Occlusion-like Syndromes in Rats and Stable Gastric Pentadecapeptide BPC 157 as Therapy

**DOI:** 10.3390/ph16060788

**Published:** 2023-05-25

**Authors:** Sanja Strbe, Ivan Maria Smoday, Ivan Krezic, Luka Kalogjera, Vlasta Vukovic, Helena Zizek, Slaven Gojkovic, Hrvoje Vranes, Ivan Barisic, Suncana Sikiric, Marijan Tepes, Katarina Oroz, Filip Brkic, Martin Drinkovic, Lidija Beketic Oreskovic, Jelena Popic, Alenka Boban Blagaic, Anita Skrtic, Mario Staresinic, Sven Seiwerth, Predrag Sikiric

**Affiliations:** 1 Department of Pharmacology, School of Medicine, University of Zagreb, 10000 Zagreb, Croatia; strbes@gmail.com (S.S.); ivansmoday1@gmail.com (I.M.S.); ivankrezic94@gmail.com (I.K.); lkalogjera9@gmail.com (L.K.); vukovic.vlasta1@gmail.com (V.V.); zizekhelena@gmail.com (H.Z.); slaven.gojkovic.007@gmail.com (S.G.); hrvoje.vranes@gmail.com (H.V.); inbarisic@gmail.com (I.B.); mtepes@gmail.com (M.T.); oroz.kat@hotmail.com (K.O.); filip.brkic777@gmail.com (F.B.); lidijabeketicoreskovic@gmail.com (L.B.O.); popic.je@gmail.com (J.P.); abblagaic@mef.hr (A.B.B.); 2 Department of Pathology, School of Medicine, University of Zagreb, 10000 Zagreb, Croatia; suncanasikiric@gmail.com (S.S.); sven.seiwerth@mef.hr (S.S.)

**Keywords:** vascular failure, neuroleptics, amphetamine, domperidone, occlusion/occlusion-like syndromes, rats, pentadecapeptide BPC 157

## Abstract

Even before behavioral disturbances, neuroleptics, amphetamine, and domperidone application rapidly emerged severe occlusion/occlusion-like syndrome, shared innate vascular and multiorgan failure in rats, comparable to occlusion/occlusion-like syndrome described with vessel(s) occlusion or similar noxious procedures application. As therapy, i.e., activation of the collateral pathways, “bypassing key” (activated azygos vein pathway, direct blood flow delivery), the stable gastric pentadecapeptide BPC 157 is a novel solution. Recently, BPC 157 therapy particularly counteracted neuroleptic- or L-NAME-induced catalepsy, lithium intoxication, and schizophrenia positive and negative symptoms (amphetamine/methamphetamine/apomorphine/ketamine). In rats with complete calvariectomy, medication (BPC 157 10 µg/kg, 10 ng/kg ip or ig) was given 5 min after distinctive dopamine agents (mg/kg ip) (haloperidol (5), fluphenazine (5), clozapine (10), risperidone (5), olanzapine (10), quetiapine (10), or aripiprazole (10), domperidone (25), amphetamine (10), and combined amphetamine and haloperidol) and assessed at 15 min thereafter. All neuroleptic-, domperidone-, and amphetamine-induced comparable vascular and multiorgan failure severe syndrome was alleviated with BPC 157 therapy as before major vessel(s) occlusion or other similar noxious procedures. Specifically, all severe lesions in the brain (i.e., immediate swelling, hemorrhage), heart (i.e., congestion, arrhythmias), and lung (i.e., congestion, hemorrhage), as well as congestion in the liver, kidney, and gastrointestinal (stomach) tract, were resolved. Intracranial (superior sagittal sinus), portal, and caval hypertension and aortal hypotension were attenuated or eliminated. BPC 157 therapy almost annihilated arterial and venous thrombosis, peripherally and centrally. Thus, rapidly acting Virchow triad circumstances that occur as dopamine central/peripheral antagonists and agonist essential class-points, fully reversed by BPC 157 therapy, might be overwhelming for both neuroleptics and amphetamine.

## 1. Introduction

We recently reviewed the brain-gut and gut-brain axes based on gut peptides’ beneficial peripheral and central effects on the internal organs and brain lesions [1]. A particular part, peripheral-central and central-peripheral link, was a rapidly progressing peripheral and central occlusion/occlusion-like syndrome, vascular and multiorgan failure in rats with major vessel occlusion or undergoing similar noxious procedures [1]. There, we emphasized the particular role of the stable gastric pentadecapeptide BPC 157 [1]. We focused on the vascular (activation of collateral pathways, depending on the given injury, counteracting occlusion/occlusion-like syndrome, vascular and multiorgan failure) [2], cytoprotective (innate epithelium and endothelium protection) [2,3,4,5,6,7], wound healing [8], and neuroprotective effect [1,9,10]. These may be essential to recover and reestablish the necessary functioning of the brain-gut and gut-brain axes [1]. There, as native and stable in human gastric juice for more than 24 h, BPC 157 appeared as a cytoprotection mediator maintaining the epithelial and endothelial integrity of the gastrointestinal tract (used in ulcerative colitis phase II, very safe and lethal dose (LD1) not achieved in toxicology studies) [1,2,3,4,5,6,7,8,9,10]. Besides, for brain-gut and gut-brain axes functioning [1], BPC 157 counteracted various encephalopathies and concomitant peripheral lesions that may appear with major intestine resection, non-steroidal anti-inflammatory drugs (NSAIDs), insulin, and neurotoxins application [1].

Here, we focused on the issue of the brain-gut axis and gut-brain axis issue functioning on a novel, likely very indicative peripheral/central link with neuroleptics, amphetamine, and domperidone. This revealed for neuroleptics, amphetamine, and domperidone a particular, rapidly progressing peripheral and central occlusion/occlusion-like syndrome as in rats with major vessel occlusion [11,12,13,14,15,16,17,18] or similar noxious procedures [19,20,21,22,23,24]. This shared occlusion/occlusion-like syndrome may be a novel common very early point even antecedent to the dopamine agents’ behavioral disturbances that BPC 157 consistently (agonists and antagonists) antagonized [25,26,27,28,29]. Likewise, this novel occlusion/occlusion-like syndrome and novel strong counteraction by the stable gastric pentadecapeptide BPC 157 therapy may become a particular target for a common therapy. 

The occlusion/occlusion-like syndrome conceptual background as a large multicausal class effect, peripheral and central was realized only quite recently [1,2,3,4,5,6,7]. Here, there was a general cytoprotective endothelial threat (i.e., a disturbed dopamine system as a long-lasting essential pitfall in cytoprotection maintenance [30,31,32,33,34,35], endothelium lesions, and thrombosis induced by neuroleptics [36,37,38,39,40,41], domperidone [42], and amphetamine [43,44,45]). This accorded a major challenge of multicausal pathology (i.e., major vessel occlusion [11,12,13,14,15,16,17,18] peripherally and centrally and similar noxious procedures [19,20,21,22,23,24] severely affecting endothelium function). With such a challenge, there was generally an innate vascular healing inability (i.e., thought to be of failed cytoprotection origin, and thereby reversed by the cytoprotective agents such as BPC 157 recovering endothelium function) [1,2,3,4,5,6,7]. The overwhelmed were small vessels. Spontaneously, they were unable to adapt and unable to take over the function of the disabled major vessels and were unable to spontaneously recover blood flow. Consequently, such occlusion/occlusion-like syndrome occurred peripherally and centrally as a severe vascular and multiorgan failure rapidly progressing with major vessel occlusion [11,12,13,14,15,16,17,18] and similar noxious procedures [19,20,21,22,23,24] severely disabling endothelium function. Commonly, it was cured with the stable gastric pentadecapeptide BPC 157 therapy “bypassing key” potential (i.e., activated collateral pathways, azygos vein direct blood delivery) (for review see, i.e., [1,2,3,4,5,6,7]). These were the lesions in the brain (intracerebral and intraventricular hemorrhage), heart (congestion and infarctions), lung (hemorrhage), congestion in the liver, kidney, and gastrointestinal tract, arrhythmias, and blood pressure disturbances (intracranial (superior sagittal sinus), portal and caval hypertension, and aortal hypotension). Arterial and venous thrombosis presented peripherally and centrally [11,12,13,14,15,16,17,18,19,20,21,22,23,24]. Together, these events tightly interconnected evidenced the crucial harmful implication of the failed major vessels, i.e., congested inferior caval vein, superior mesenteric vein, and collapsed azygos vein (no direct blood flow, no rescuing pathway). Rapidly acting Virchow triad circumstances [11,12,13,14,15,16,17,18,19,20,21,22,23,24] rapidly developed the dysfunctional brain-gut and gut-brain axes in occlusion/occlusion-like syndromes whatever the cause [1,2,3,4,5,6,7]. On the other hand, these were all attenuated/eliminated by BPC 157 therapy [11,12,13,14,15,16,17,18,19,20,21,22,23,24]. Multiple improved outcomes determined the roles of the activated collateral pathways. For BPC 157 therapy, the indicated azygos vein direct blood delivery is resolving a simultaneous pleiotropic beneficial effect that may take place, simultaneously peripherally and centrally [11,12,13,14,15,16,17,18,19,20,21,22,23,24]. The counteraction of occlusion/occlusion-like syndrome peripheral and central, and vice versa, as a whole means a particular role in showing the reestablished functioning of the brain-gut and gut-brain axes has recently been fully emphasized [1,2,3,4,5,6,7].

These peripheral-central interconnected associations may be interesting in terms of the dopamine- and nitric oxide (NO)-system’s essential significance in stomach cytoprotection and central nervous system disorders [1]. Namely, the BPC 157 particular therapy counteracted the neuroleptic- or N(G)-nitro-L-arginine methylester (L-NAME)-induced catalepsy, as well as the schizophrenia positive and negative symptoms (amphetamine, apomorphine, methamphetamine, ketamine) [25,26,27,28,29]. These occurred in particular relation to NO-agents (L-NAME and/or L-arginine) effects [25,26,27,28,29]. Likewise was an occurrence in the periphery. BPC 157 counteracted dopamine disturbances (i.e., gastric lesions, sphincter failure [46,47,48], and arrhythmias [49] induced by neuroleptics). Also, BPC 157 therapy counteracted NO-agents-induced disturbances (i.e., hypertension, hypotension, pro-thrombotic and anti-thrombotic effects) [50,51,52,53,54]. Thereby, this therapy’s central relevance for the neuroleptics, amphetamine, and domperidone activities (i.e., counteraction of the brain swelling, counteraction of intracranial (superior sagittal sinus) hypertension) should be the counteraction of the whole occlusion/occlusion-like syndrome. In particular, this should be the recruitment of the collateral rescuing pathways, i.e., azygos vein direct blood flow delivery [1,2,3,4,5,6,7]. Of note, with reference to psychoactive agents and BPC 157 counteraction potential, the principle of the counteraction of the whole occlusion/occlusion-like syndrome, via recovering of the azygos vein rescuing pathway, direct blood flow delivery, was already shown with full counteraction of the lithium intoxication [20]. A similar occlusion/occlusion-like syndrome and an equal outcome appeared with counteraction of intoxication with absolute alcohol [23]. 

Finally, this contention might bring into the cytoprotection issue and novel common therapy solution the disturbances induced by the various typical and atypical neuroleptics, amphetamine, acting centrally and peripherally, and peripherally acting prokinetic domperidone. Confirming these points toward malfunctioning brain-gut axis and gut-brain axis [1] as class adverse effects required the consistent effect of several prototypic neuroleptics (i.e., haloperidol, fluphenazine, clozapine, olanzapine, risperidone, quetiapine, and aripiprazole) vs. amphetamine (and combined amphetamine and haloperidol) vs. domperidone (central vs. peripheral effect). 

Thereby, we suggest that with the congruent effect of all of these agents, the common neuroleptic-amphetamine-domperidone-occlusion/occlusion-like syndrome would be established. If so, BPC 157 therapy, given either intraperitoneally or intragastrically (as a part of its mentioned cytoprotection agenda) [1,2,3,4,5,6,7], in the µg-ng regimen as applied before in previous vascular studies [11,12,13,14,15,16,17,18,19,20,21,22,23,24] should have a strong beneficial effect. This may be the rapid upgrading of the small vessel (i.e., azygos vein), substituting the function of the disabled major vessel, and re-establishing the reorganized blood flow [1,2,3,4,5,6,7]. This should result in the attenuation or elimination of occlusion/occlusion-like syndrome. This should be a possible clue for BPC 157 resolving interaction with mentioned dopamine agents [25,26,27,28,29], dopamine and NO-system, and therapy potential [1,2,3,4,5,6,7].

## 2. Results

Intracranial (superior sagittal sinus) hypertension and aortal hypotension, major ECG disturbances, progressing arterial and vein thrombosis, and lesions in the brain, heart, lungs, liver, kidneys, and gastrointestinal tract appeared after the application of the overdose of the distinctive dopamine agents, antagonists, central (antipsychotics) and peripheral (prokinetic domperidone), and agonist (amphetamine). We revealed this injurious course as provoked rapid vascular failure and shared perilous syndrome occurring peripherally and centrally. Indeed, unless BPC 157 therapy was given, in all dopamine agent regimens (haloperidol, fluphenazine, clozapine, risperidone, olanzapine, quetiapine, aripiprazole, domperidone, amphetamine, and the combination of amphetamine and haloperidol), there was a shared noxious syndrome. The noted syndrome was similar to those previously described in vascular failure studies (occlusion/occlusion-like syndromes, with major vessel occlusion or with other similar noxious procedures, endothelium damaging agent application, myocardial infarction, acute pancreatitis, and intra-abdominal hypertension) [14,15,16,18,20,21,22,23,24].

The stable gastric pentadecapeptide BPC 157 therapy application’s common key finding might be the prompt and sustained activation of the azygos vein in BPC-157-treated rats. The direct blood delivery via the azygos vein from the inferior caval vein to the superior caval vein might instantly break the injurious circle. Consequently, there was a counteraction of the adjacent adverse syndrome (i.e., attenuated/counteracted intracranial (superior sagittal sinus) hypertension and aortal hypotension; major ECG disturbances; progressing arterial and vein thrombosis; lesions in the brain, heart, lungs, liver, kidneys, and gastrointestinal tract). This BPC 157 therapy effect was comparable to the previous BPC 157 therapy of the mentioned occlusion/occlusion-like syndromes [14,15,16,18,20,21,22,23,24], i.e., a similar therapeutic effect of the used regimens (intraperitoneal and intragastric) and applied dosage range (µg–ng). The similar effects of the intragastric BPC 157 application in haloperidol and amphetamine rats were not specifically shown.

### 2.1. A Perilous Syndrome Occurred Peripherally and Centrally

#### 2.1.1. Blood Pressure Disturbances

As a cause–consequence relation indicative of the effectiveness of the therapy application, we emphasized the blood pressure disturbances induced by the neuroleptics, domperidone, and amphetamine, all of which BPC 157 might reduce consistently. These were otherwise induced rapidly by haloperidol, fluphenazine, clozapine, risperidone, olanzapine, quetiapine, aripiprazole, domperidone, amphetamine, and the combination of amphetamine and haloperidol presented peripherally (portal and caval hypertension, aortal hypotension) as well as even more centrally (superior sagittal sinus hypertension) (Table 1). Rapidly presented, the portal and caval hypertension, and even more the intracranial (superior sagittal sinus) hypertension and the aortal hypotension, were eliminated or markedly attenuated by BPC 157 application. Furthermore, in the rats challenged with amphetamine as well as in the rats challenged with haloperidol, there was no difference between the BPC 157 therapy applied intragastrically and BPC 157 therapy applied intraperitoneally, providing closely corresponding beneficial effects (data not specifically shown).

#### 2.1.2. Thrombosis

Likewise, with BPC 157 therapy, prompt reduction of thrombosis appeared both peripherally and centrally (Table 1). This might indicate the effective cause–consequence course of the therapy both peripherally and centrally. Without BPC 157 therapy, with all given dopamine agents, as a shared effect of the haloperidol, fluphenazine, clozapine, risperidone, olanzapine, quetiapine, aripiprazole, domperidone, amphetamine, and amphetamine and haloperidol combination, thrombosis progressed peripherally in veins (i.e., portal vein and inferior caval vein) as well as in arteries (i.e., abdominal aorta) and centrally (i.e., superior sagittal sinus). Furthermore, the rats challenged with amphetamine and those challenged with haloperidol showed no difference between the beneficial effect of the BPC 157 therapy applied intragastrically and BPC 157 therapy applied intraperitoneally (data not specifically shown).

#### 2.1.3. Collateral Pathways, Blood Vessels, and Gross Brain Presentation

**Figure 1:**
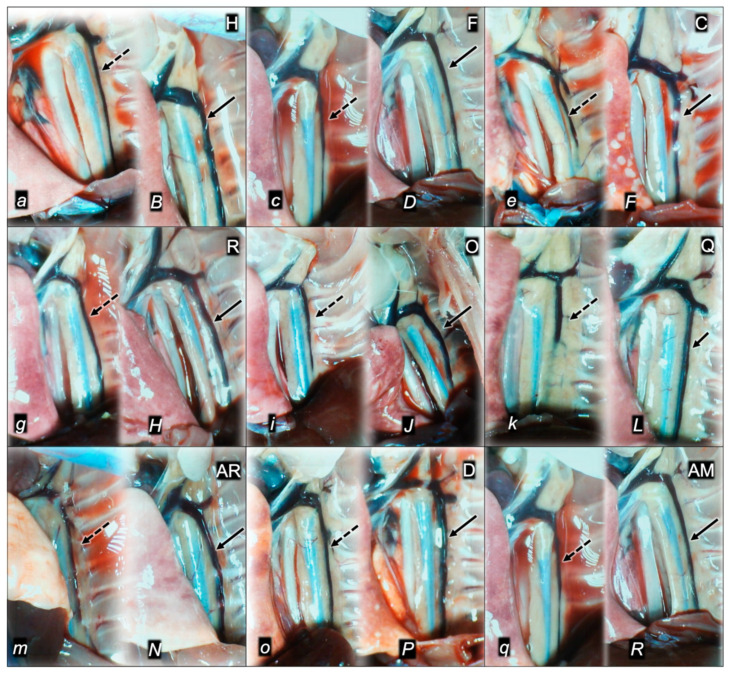
Illustrative presentation of azygos vein in control rats (*italic small letters*, dashed arrows) and in BPC 157 treated rats (*italic capital letters*, full arrows) at 15 min following application of haloperidol (H) (*a, B*), fluphenazine (F) (*c, D*), clozapine (C) (*e, F*), risperidone (R) (*g, H*), olanzapine (O) (*i, J*), quetiapine (Q) (*k, L*), aripiprazole (AR) (*m, N*), domperidone (D) (*o, P*), and amphetamine (AM) (*q, R*).

**Figure 2:**
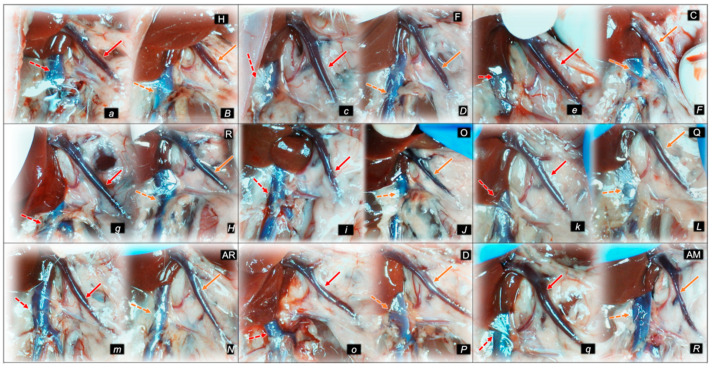
Illustrative presentation of the control rats (*italic small letters*) and of the BPC 157-treated rats (*italic capital letters*) of the superior mesenteric vein (full arrows, red (controls), yellow (BPC 157)) and inferior caval vein (dashed arrows, red (controls), yellow (BPC 157)) at 15 min following application of haloperidol (H) (*a, B*), fluphenazine (F) (*c, D*), clozapine (C) (*e, F*), risperidone (R) (*g, H*), olanzapine (O) (*i, J*), quetiapine (Q) (*k, L*), aripiprazole (AR) (*m, N*), domperidone (D) (*o, P*), and amphetamine (AM) (*q, R*).

**Figure 3:**
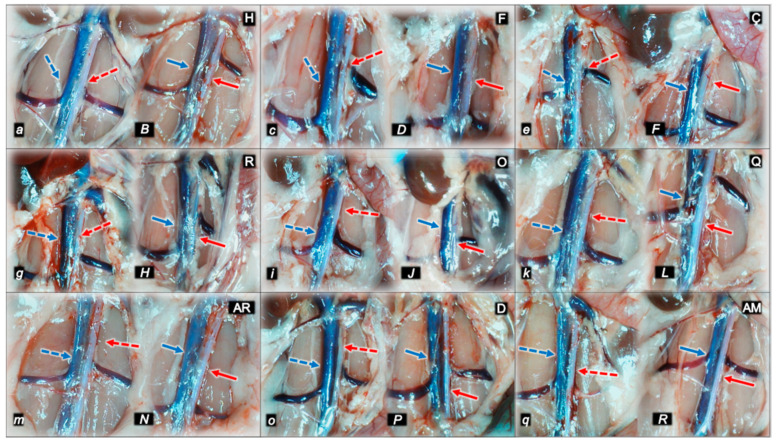
Illustrative presentation of inferior caval vein (blue arrows) and abdominal aorta (red arrows) in the control rats (*italic small letters*, dashed arrows) and in the BPC-157-treated rats (*italic capital letters*, full arrows) at 15 min following application of haloperidol (H) (*a, B*), fluphenazine (F) (*c, D*), clozapine (C) (*e, F*), risperidone (R) (*g, H*), olanzapine (O) (*i, J*), quetiapine (Q) (*k, L*), aripiprazole (AR) (*m, N*), domperidone (D) (*o, P*), and amphetamine (AM) (*q, R*).

Indicatively for a common clue (activation of collateral pathways) that might not be functioning, all of the dopamine agents (haloperidol, fluphenazine, clozapine, risperidone, olanzapine, quetiapine, aripiprazole, domperidone, amphetamine, and amphetamine and haloperidol combination) converge to the similar disturbances. We used the disturbed presentation of the azygos vein, superior mesenteric vein, inferior caval vein, and abdominal aorta as indicative illustrations (Figure 1, Figure 2 and Figure 3). Thereby, in general (providing the similar effects of the otherwise distinctive dopamine agents, antagonists, and central antipsychotics) and peripheral (prokinetic domperidone), and agonist (amphetamine)), as well as in particular (considering each of the given agents), we might envisage the failed collateral pathway presentation (Figure 1, Figure 2 and Figure 3). Contrarily, with BPC 157 therapy, advanced collateral pathway presentation (Figure 1, Figure 2 and Figure 3) occurred. Evidently, it was a follow-up to the attenuation of blood pressure disturbances, peripherally and centrally, progressing thrombosis fully counteracted in all vessels investigated, veins and arteries, peripherally and centrally (Table 1), the particular vessels’ recruitment that may compensate for the major vessel failure and blood stasis seen peripherally and centrally. The similar effects of intragastric BPC 157 application in the amphetamine and haloperidol rats are not specifically shown. 

**Figure 4:**
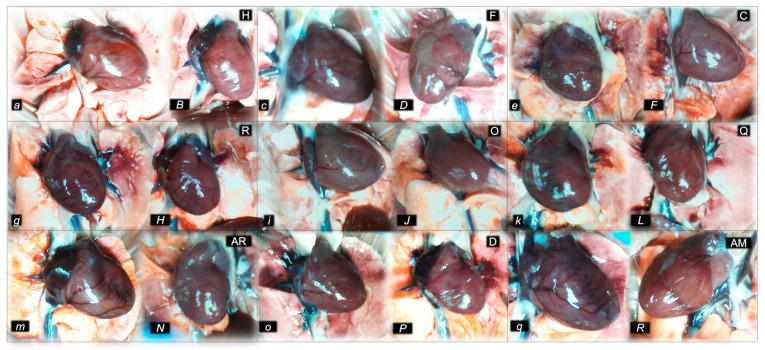
Illustrative presentation of the heart in the control rats (*italic small letters*) and in the BPC 157 treated rats (*italic capital letters*) at 15 min following application of haloperidol (H) (*a, B*), fluphenazine (F) (*c, D*), clozapine (C) (*e, F*), risperidone (R) (*g, H*), olanzapine (O) (*i, J*), quetiapine (Q) (*k, L*), aripiprazole (AR) (*m, N*), domperidone (D) (*o, P*), and amphetamine (AM) (*q, R*).

**Figure 5:**
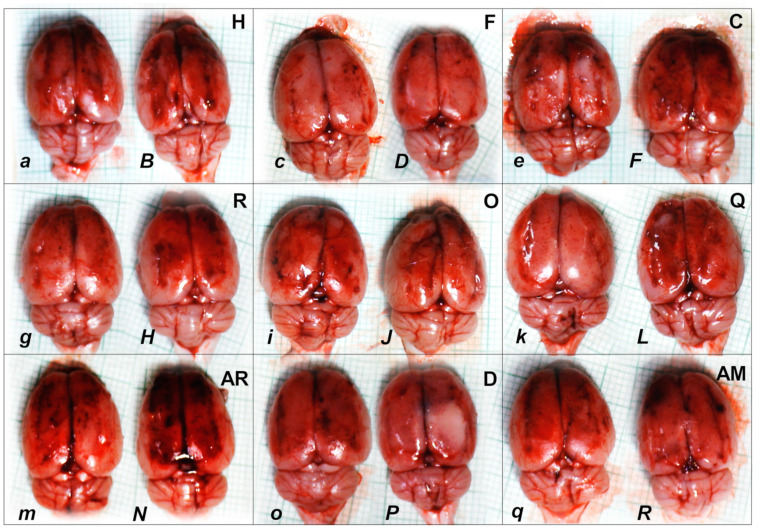
Illustrative presentation of the brain in the control rats (*italic small letters*) and in the BPC 157 treated rats (*italic capital letters*) at 15 min following application of haloperidol (H) (*a, B*), fluphenazine (F) (*c, D*), clozapine (C) (*e, F*), risperidone (R) (*g, H*), olanzapine (O) (*i, J*), quetiapine (Q) (*k, L*), aripiprazole (AR) (*m, N*), domperidone (D) (*o, P*), and amphetamine (AM) (*q, R*).

Consequently, the activated defensive response may be summarized with the particular effects of BPC 157 on the relative volume (Table 2). Otherwise, without therapy, the regular outcome was congested vessels (superior mesenteric vein and inferior caval vein) (Figure 2 and Figure 3) (because of the trapped volume, congested liver and lung), dilated heart, collapsed vessels (non-functioning azygos vein), and swollen brain (Figure 1, Figure 2 and Figure 3). BPC 157 might decrease the increased relative volume of the superior mesenteric vein and inferior caval vein (congestion) (Table 2, Figure 2 and Figure 3). The failed volume of the azygos vein was reversed (i.e., increased as the azygos vein was reactivated) (Table 2, Figure 1). Consistently, BPC 157 therapy might bring these vessels (Figure 1, Figure 2 and Figure 3) and heart presentation (Figure 4) close to a normal vessel and heart presentation and close to normal functioning to reestablish blood flow (multiorgan lesions largely attenuated). As additional proof, there is brain swelling in all rats not given therapy (Figure 5), increased intracranial (superior sagittal sinus) hypertension, and increased volume (associated with considerable brain injuries) (Figure 4) that BPC 157 administration rapidly counteracted, inducing a considerable decrease toward normal brain presentation (Figure 5) and negative pressure values (Table 1). In addition, in the rats challenged with amphetamine as well as in the rats challenged with haloperidol, the BPC 157 therapy applied intragastrically provided beneficial effects closely corresponding to the intraperitoneal regimen (data not specifically shown).

#### 2.1.4. Heart and ECG Disturbances

Commonly, the outcome with haloperidol, fluphenazine, clozapine, risperidone, olanzapine, quetiapine, aripiprazole, and domperidone was continuous tachycardia along with prolonged PQ and QTc intervals (Table 3). With amphetamine, the outcome was tachycardia and prolonged PQ intervals but short QTc intervals (Table 3). As a likely specific effect considering the effect on the whole syndrome (lacking mutual antagonization), amphetamine and haloperidol, given as dopamine agonist and dopamine antagonist combined, might antagonize each other ECG-disturbances (prolonged PQ-intervals, and prolonged QTc-interval (haloperidol) and short QTc-interval (amphetamine), while tachycardia remained unopposed. On the other hand, the counteracting evidence demonstrated that in all BPC-157-treated rats, tachycardia, QTc intervals, or PQ intervals were regularly attenuated or absent. This occurred along with a counteraction of myocardial congestion (Figure 4). In addition, in the rats challenged with amphetamine as well as in the rats challenged with haloperidol, the BPC 157 therapy applied intragastrically demonstrated similar beneficial effects as the intraperitoneal regimen (data not specifically shown).

### 2.2. A Perilous Syndrome Occurred Peripherally

#### 2.2.1. Gastrointestinal, Lung, Liver, Kidney, and Heart Lesions

**Figure 6:**
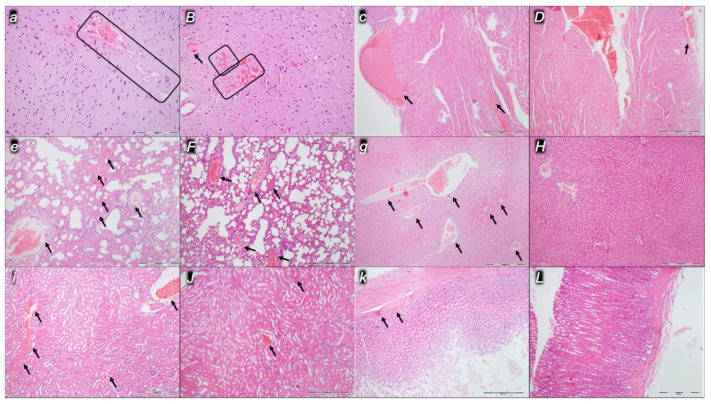
Haloperidol-rats. Illustrative microscopic presentation of the brain (*a*, *B*), heart (*c*, *D*), lung (*e*, *F*), liver (*g*, *H*), kidney (*i*, *J*), and stomach (*k*, *L*) in the saline-treated rats (control, small italic letters) and BPC 157-treated rats (capital italic letters) at the end of the experiments at the 15 min. Brain (*a*, *B*). Control (*a*). A pronounced edema and congestion were visible affecting the cerebrum, and more prominent intracerebral cortical hemorrhage involving larger areas of cerebral brain tissue affecting the neocortex (rectangle area). BPC 157 (*B*). Only mild edema and congestion were found, with small, focal, and superficial areas of neocortical hemorrhage (rectangle areas and black arrow). Heart (*c*, *D*). Control (*c*). Moderated myocardial congestion (black arrows). BPC 157 (*D*). Only mild myocardial congestion (black arrow). Lung (*e*, *F*). Control (*e*). Moderate congestion of the lung parenchyma (black arrows). BPC 157 (*F*). Only mild lung congestion (black arrows). Liver (*g*, *H*). Control (*g*). A moderate dilatation and congestion of blood vessels in the portal tracts, central veins, and sinusoids (black arrows). BPC 157 (*H*). No changes were found. Kidney (*i*, *J*). Control (*i*). A moderate dilatation of blood vessels and congestion in the kidney tissue, as well as glomeruli, was found (black arrows). BPC 157 (*J*). Mild dilatation of blood vessels and congestion in the kidney tissue, as well as glomeruli (black arrows). Gastrointestinal lesion (*k*, *L*). Control (*k*). Moderate congestion of the stomach wall (black arrows). BPC 157 (*L*). No changes were found in the stomach wall. HE; magnification 100× (*c*, *D*, *e*, *F*, *g*, *H*, *i*, *J*, *k*, *L*), magnification 200× (*a*, *B*).

**Figure 7:**
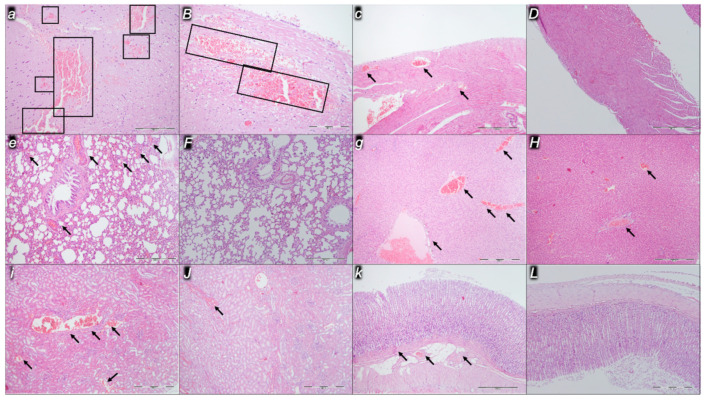
Fluphenazine-rats. Illustrative microscopic presentation of the brain (*a*, *B*), heart (*c*, *D*), lung (*e*, *F*), liver (*g*, *H*), kidney (*i*, *J*), and stomach (*k*, *L*) in the saline-treated rats (control, small italic letters) and BPC 157-treated rats (capital italic letters) at the end of the experiments at the 15 min. Brain (*a*, *B*). Control (*a*). A pronounced edema and congestion were visible affecting the cerebrum, and more prominent intracerebral cortical hemorrhage involving larger areas of cerebral brain tissue affecting the neocortex (rectangle areas). BPC 157 (*B*). Only mild edema and congestion were found, with small, focal, and superficial areas of neocortical hemorrhage (rectangle areas). Heart (*c*, *D*). Control (*c*). Marked myocardial congestion (black arrows). BPC 157 (*D*). No changes were found. Lung (*e*, *F*). Control (*e*). Moderate congestion of the lung parenchyma (black arrows). BPC 157 (*F*). No changes were found. Liver (*g*, *H*). Control (*g*). Marked dilatation and congestion of blood vessels in the portal tracts, central veins, and sinusoids (black arrows). BPC 157 (*H*). Only a mild dilatation and congestion of blood vessels in the portal tracts (black arrows). Kidney (*i*, *J*). Control (*i*). A moderate dilatation of blood vessels and congestion in the kidney tissue, as well as glomeruli, was found (black arrows). BPC 157 (*J*). Mild dilatation of blood vessels and congestion in the kidney tissue, as well as glomeruli (black arrows). Gastrointestinal lesion (*k*, *L*). Control (*k*). Mild congestion of the stomach wall(black arrows). BPC 157 (*L*). No changes were found in the stomach wall. HE; magnification 100× (*c*, *D*, *e*, *F*, *g*, *H*, *i*, *J*, *k*, *L*), magnification 200× (*a*, *B*).

**Figure 8:**
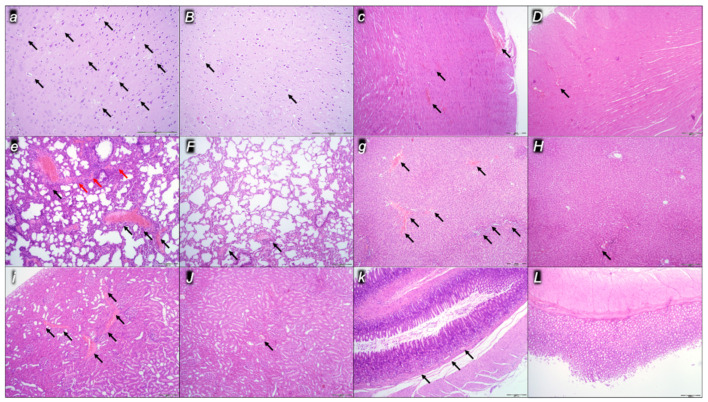
Clozapine-rats. Illustrative microscopic presentation of the brain (*a*, *B*), heart (*c*, *D*), lung (*e*, *F*), liver (*g*, *H*), kidney (*i*, *J*), and stomach (*k*, *L*) in the saline-treated rats (control, small italic letters) and BPC 157-treated rats (capital italic letters) at the end of the experiments at the 15 min. Brain (*a*, *B*). Control (*a*). A pronounced edema (black arrows) and congestion were visible in the brain tissue, increased number of affected karyopyknotic cells in the cerebral cortex. BPC 157 (*B*). These were attenuated in BPC 157 treated rats (black arrows). Heart (*c*, *D*). Control (*c*). Moderate myocardial congestion (black arrows). BPC 157 (*D*). Mild myocardial congestion was found (black arrow). Lung (*e*, *F*). Control (*e*). Marked congestion (black arrows) of the lung parenchyma with intra-alveolar hemorrhage (red arrows). BPC 157 (*F*). Only mild lung congestion was found (black arrows). Liver (*g*, *H*). Control (*g*). A marked dilatation and congestion of blood vessels in the portal tracts, central veins, and sinusoids (black arrows). BPC 157 (*H*). Only a mild dilatation and congestion of blood vessels in the portal tracts (black arrow). Kidney (*i*, *J*). Control (*i*). A moderate dilatation of blood vessels and congestion in the kidney tissue, as well as glomeruli, was found (black arrows). BPC 157 (*J*). Mild dilatation of blood vessels and congestion in the kidney tissue, as well as glomeruli (black arrow). Gastrointestinal lesion (*k*, *L*). Control (*k*). Mild congestion of the stomach wall (black arrows). BPC 157 (*L*). No changes were found in the stomach wall. HE; magnification 100× (*c*, *D*, *e*, *F*, *g*, *H*, *i*, *J*, *k*, *L*), magnification 200× (*a*, *B*).

**Figure 9:**
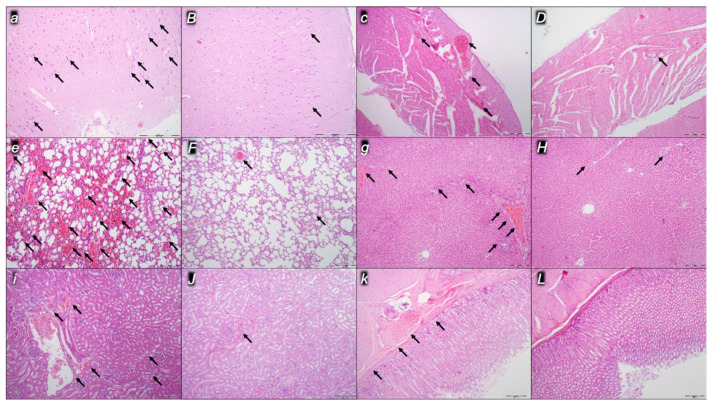
Risperidone-rats. Illustrative microscopic presentation of the brain (*a*, *B*), heart (*c*, *D*), lung (*e*, *F*), liver (*g*, *H*), kidney (*i*, *J*), and stomach (*k*, *L*) in the saline-treated rats (control, small italic letters) and BPC 157-treated rats (capital italic letters) at the end of the experiments at the 15 min. Brain (*a*, *B*). Control (*a*). A pronounced edema and congestion were visible in the brain tissue, increased number of karyopyknotic cells affecting the cerebral cortex (black arrows). BPC 157 (*B*). These were attenuated in BPC 157 treated rats (black arrows). Heart (*c*, *D*). Control (*c*). Moderate myocardial congestion (black arrows). BPC 157 (*D*). Mild myocardial congestion (black arrow). Lung (*e*, *F*). Control (*e*). A marked congestion of the lung parenchyma with intra-alveolar hemorrhage (black arrows). BPC 157 (*F*). Only mild lung congestion was found (black arrow). Liver (*g*, *H*). Control (*g*). A marked dilatation and congestion of blood vessels in the portal tracts, central veins, and sinusoids (black arrows). BPC 157 (*H*). Only a mild dilatation and congestion of blood vessels in the portal tracts (black arrows). Kidney (*i*, *J*). Control (*i*). A moderate dilatation of blood vessels and congestion in the kidney tissue, as well as glomeruli, was found (black arrows). BPC 157 (*J*). Mild dilatation of blood vessels and congestion in the kidney tissue, as well as glomeruli (black arrow). Gastrointestinal lesion (*k*, *L*). Control (*k*). Moderate congestion of the stomach wall (black arrows). BPC 157 (*L*). No changes were found in the stomach wall. HE; magnification 100× (*c*, *D*, *e*, *F*, *g*, *H*, *i*, *J*, *k*, *L*), magnification 200× (*a*, *B*).

**Figure 10:**
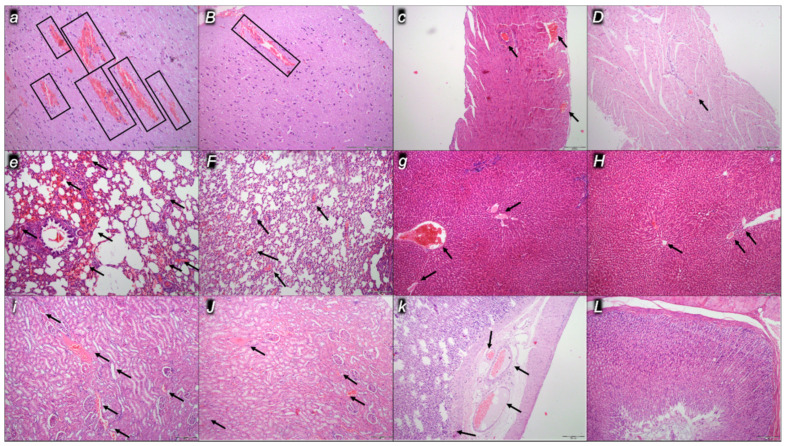
Olanzapine-rats. Illustrative microscopic presentation of the brain (*a*, *B*), heart (*c*, *D*), lung (*e*, *F*), liver (*g*, *H*), kidney (*i*, *J*), and stomach (*k*, *L*) in the saline-treated rats (control, small italic letters) and BPC 157-treated rats (capital italic letters) at the end of the experiments at the 15 min. Brain (*a*, *B*). Control (*a*). A pronounced edema and congestion were visible in the brain tissue, an increased number of karyopyknotic cells affecting the cerebral cortex, and a prominent intracerebral cortical hemorrhage involving larger areas of brain tissue with transmural involvement of the neocortex (rectangular areas). BPC 157 (*B*). These were attenuated in BPC 157 treated rats. Only the superficial half of neocortical layers are affected by hemorrhage (rectangular area). Heart (*c*, *D*). Control (*c*). Moderate myocardial congestion (black arrows). BPC 157 (*D*). Mild myocardial congestion was found (black arrow). Lung (*e*, *F*). Control (*e*). Moderate congestion of the lung parenchyma (black arrows). BPC 157 (*F*). Only mild lung congestion was found (black arrows). Liver (*g*, *H*). Control (*g*). Marked dilatation and congestion of blood vessels in the portal tracts, central veins, and sinusoids (black arrows). BPC 157 (*H*). Only a mild dilatation and congestion of blood vessels in the portal tracts (black arrows). Kidney (*i*, *J*). Control (*i*). Moderate dilatation of blood vessels and congestion in the kidney tissue, as well as glomeruli, was found (black arrows). BPC 157 (*J*). Mild dilatation of blood vessels and congestion in the kidney tissue, as well as glomeruli (black arrows). Gastrointestinal lesion (*k*, *L*). Control (*k*). Moderate congestion of the stomach wall (black arrows). BPC 157 (*L*). No changes were found in the stomach wall. HE; magnification 100× (*c*, *D*, *e*, *F*, *g*, *H*, *i*, *J*, *k*, *L*), magnification 200× (*a*, *B*).

**Figure 11:**
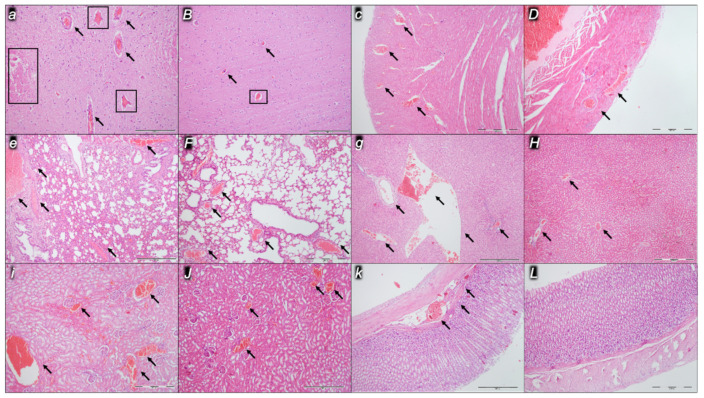
Quetiapine-rats. Illustrative microscopic presentation of the brain (*a*, *B*), heart (*c*, *D*), lung (*e*, *F*), liver (*g*, *H*), kidney (*i*, *J*), and stomach (*k*, *L*) in the saline-treated rats (control, small italic letters) and BPC 157-treated rats (capital italic letters) at the end of the experiments at the 15 min. Brain (*a*, *B*). Control (*a*). A pronounced edema and congestion were visible affecting the cerebrum (black arrows), and more prominent intracerebral cortical hemorrhage involving larger areas of cerebral brain tissue affecting the transmural neocortex (rectangular areas). BPC 157 (*B*). Only mild edema and congestion were found (black arrows), with small, focal, and superficial areas of neocortical hemorrhage (rectangular area). Heart (*c*, *D*). Control (*c*). Marked myocardial congestion (black arrows). BPC 157 (*D*). Moderate myocardial congestion (black arrows). Lung (*e*, *F*). Control (*e*). Moderate congestion of the lung parenchyma (black arrows). BPC 157 (*F*). Only mild lung congestion (black arrows). Liver (*g*, *H*). Control (*g*). A marked dilatation and congestion of blood vessels in the portal tracts, central veins, and sinusoids (black arrows). BPC 157 (*H*). Only a mild dilatation and congestion of blood vessels in the portal tracts (black arrows). Kidney (*i*, *J*). Control (*i*). A marked dilatation of blood vessels and congestion in the kidney tissue, as well as glomeruli, was found (black arrows). BPC 157 (*J*). Mild dilatation of blood vessels and congestion in the kidney tissue, as well as glomeruli (black arrows). Gastrointestinal lesion (*k*, *L*). Control (*k*). Moderate congestion of the stomach wall (black arrows). BPC 157 (*L*). No changes were found in the stomach wall. HE; magnification 100× (*c*, *D*, *e*, *F*, *g*, *H*, *i*, *J*, *k*, *L*), magnification 200× (*a*, *B*).

**Figure 12:**
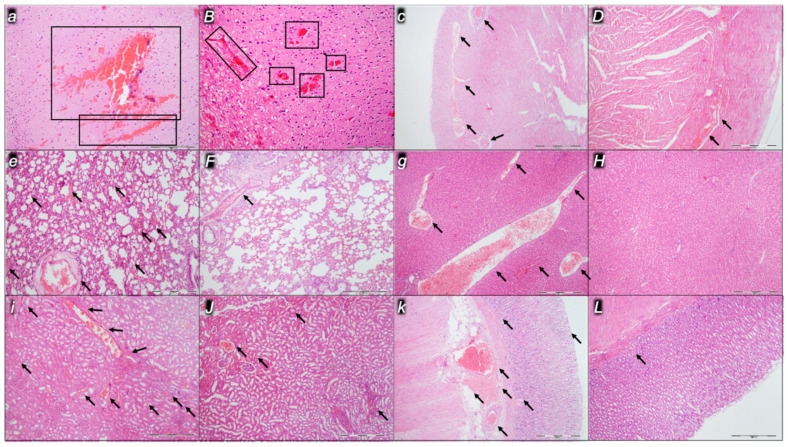
Aripiprazole-rats. Illustrative microscopic presentation of the brain (*a*, *B*), heart (*c*, *D*), lung (*e*, *F*), liver (*g*, *H*), kidney (*i*, *J*), and stomach (*k*, *L*) in the saline-treated rats (control, small italic letters) and BPC 157-treated rats (capital italic letters) at the end of the experiments at the 15 min. Brain (*a*, *B*). Control (*a*). A pronounced edema and congestion were visible affecting the cerebrum, and more prominent intracerebral cortical hemorrhage involving larger areas of cerebral brain tissue affecting the neocortex (rectangular areas). BPC 157 (*B*). Only mild edema and congestion were found, with small, focal, and superficial areas of neocortical hemorrhage (rectangular areas). Heart (*c*, *D*). Control (*c*). Moderated myocardial congestion (black arrows). BPC 157 (*D*). Only mild myocardial congestion (black arrows). Lung (*e*, *F*). Control (*e*). Moderate congestion of the lung parenchyma with intra-alveolar hemorrhage (black arrows). BPC 157 (*F*). Only mild lung congestion (black arrow). Liver (*g*, *H*). Control (*g*). A marked dilatation and congestion of blood vessels in the portal tracts, central veins, and sinusoids (black arrows). BPC 157 (*H*). No changes were found. Kidney (*i*, *J*). Control (*i*). A marked dilatation of blood vessels and congestion in the kidney tissue, as well as glomeruli, was found (black arrows). BPC 157 (*J*). Mild dilatation of blood vessels and congestion in the kidney tissue, as well as glomeruli (black arrows). Gastrointestinal lesion (*k*, *L*). Control (*k*). Moderate congestion of the stomach wall (black arrows). BPC 157 (*L*). No changes were found in the stomach wall. HE; magnification 100× (*c*, *D*, *e*, *F*, *g*, *H*, *i*, *J*, *k*, *L*), magnification 200× (*a*, *B*).

**Figure 13:**
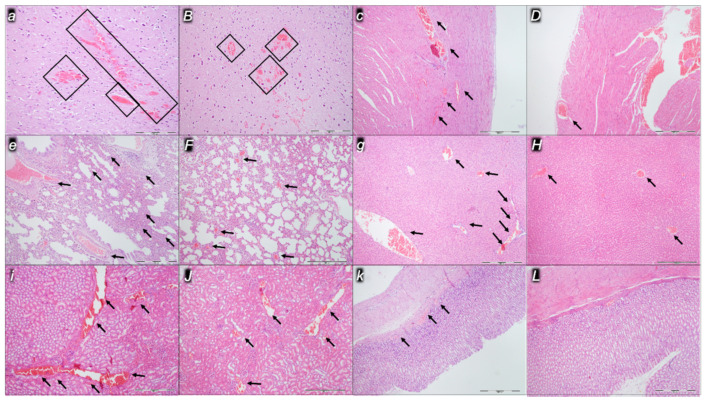
Domperidone-rats. Illustrative microscopic presentation of the brain (*a*, *B*), heart (*c*, *D*), lung (*e*, *F*), liver (*g*, *H*), kidney (*i*, *J*), and stomach (*k*, *L*) in the saline-treated rats (control, small italic letters) and BPC 157-treated rats (capital italic letters) at the end of the experiments at the 15 min. Brain (*a*, *B*). Control (*a*). A pronounced edema and congestion were visible affecting the cerebrum, and more prominent intracerebral cortical hemorrhage involving larger areas of cerebral brain tissue affecting the neocortex (rectangular areas). BPC 157 (*B*). Only mild edema and congestion were found, with small, focal, and superficial areas of neocortical hemorrhage (rectangular areas). Heart (*c*, *D*). Control (*c*). Marked myocardial congestion (black arrows). BPC 157 (*D*). Only mild myocardial congestion (black arrow). Lung (*e*, *F*). Control (*e*). Moderate congestion of the lung parenchyma (black arrows). BPC 157 (*F*). Only mild lung congestion (black arrows). Liver (*g*, *H*). Control (*g*). A moderate dilatation and congestion of blood vessels in the portal tracts, central veins, and sinusoids (black arrows). BPC 157 (*H*). Mild congestion was found (black arrows). Kidney (*i*, *J*). Control (*i*). Marked dilatation of blood vessels and congestion in the kidney tissue, as well as glomeruli, was found (black arrows). BPC 157 (*J*). Mild dilatation of blood vessels and congestion in the kidney tissue, as well as glomeruli (black arrows). Gastrointestinal lesion (*k*, *L*). Control (*k*). Moderate congestion of the stomach wall (black arrows). BPC 157 (*L*). No changes were found in the stomach wall. HE; magnification 100× (*c*, *D*, *e*, *F*, *g*, *H*, *i*, *J*, *k*, *L*), magnification 200× (*a*, *B*).

**Figure 14:**
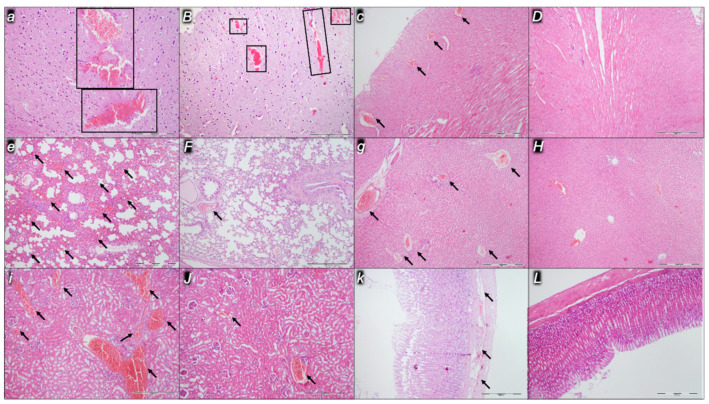
Amphetamine-rats. Illustrative microscopic presentation of the brain (*a*, *B*), heart (*c*, *D*), lung (*e*, *F*), liver (*g*, *H*), kidney (*i*, *J*), and stomach (*k*, *L*) in the saline-treated rats (control, small italic letters) and BPC 157-treated rats (capital italic letters) at the end of the experiments at the 15 min. Brain (*a*, *B*). Control (*a*). A pronounced edema and congestion were visible affecting the cerebrum, and more prominent intracerebral cortical hemorrhage involving larger areas of cerebral brain tissue affecting the neocortex (rectangular areas). BPC 157 (*B*). Only mild edema and congestion were found, with small, focal, and superficial areas of neocortical hemorrhage (rectangular areas). Heart (*c*, *D*). Control (*c*). Moderated myocardial congestion (black arrows). BPC 157 (*D*). No changes were found. Lung (*e*, *F*). Control (*e*). A marked congestion of the lung parenchyma with intra-alveolar hemorrhage (black arrows). BPC 157 (*F*). Only mild lung congestion (black arrow). Liver (*g*, *H*). Control (*g*). A moderate dilatation and congestion of blood vessels in the portal tracts, central veins, and sinusoids (black arrows). BPC 157 (*H*). No changes were found. Kidney (*i*, *J*). Control (*i*). A marked dilatation of blood vessels and congestion in the kidney tissue, as well as glomeruli, was found (black arrows). BPC 157 (*J*). Mild dilatation of blood vessels and congestion in the kidney tissue, as well as glomeruli (black arrows). Gastrointestinal lesion (*k*, *L*). Control (*k*). Mild congestion of the stomach wall (black arrows). BPC 157 (*L*). No changes were found in the stomach wall. HE; magnification 100× (*c*, *D*, *e*, *F*, *g*, *H*, *i*, *J*, *k*, *L*), magnification 200× (*a*, *B*).

Indicatively for a common clue that might be failing (i.e., intracranial (superior sagittal sinus), portal, and caval hypertension; aortal hypotension; progressed thrombosis, peripherally and centrally; failed collateral recruitment), all the dopamine agents (haloperidol, fluphenazine, clozapine, risperidone, olanzapine, quetiapine, aripiprazole, domperidone, amphetamine, and the combination of amphetamine and haloperidol) converge to the similar organ lesion. Commonly noted to be consistently expressed in untreated rats, the particular lesion evidence in the affected organs may indicate particularity in the lesion course (Table 4) with the given agent, haloperidol (Figure 6), fluphenazine (Figure 7), clozapine (Figure 8), risperidone (Figure 9), olanzapine (Figure 10), quetiapine (Figure 11), aripiprazole (Figure 12), domperidone (Figure 13), or amphetamine (Figure 14). Thereby, the reduced severity of lesions by BPC 157 therapy may be seen as part of the cause–consequence therapeutic course along with the reduced intracranial (superior sagittal sinus), portal, and caval hypertension, reduced aortal hypotension, and immediate impact of the activated collateral pathway. In addition, in the rats challenged with amphetamine as well as in the rats challenged with haloperidol, similar beneficial effects were noted with BPC 157 therapy applied intragastrically (data not specifically shown).

#### 2.2.2. Heart

Considerable myocardial congestion was commonly noted in all the control rats, in particular in those challenged with haloperidol, fluphenazine, clozapine, and quetiapine. Contrarily, in rats treated with BPC 157, these lesions were markedly attenuated or even completely annihilated in rats challenged with fluphenazine, amphetamine, and amphetamine combined with haloperidol.

#### 2.2.3. Lung

Considerable lung parenchyma congestion was commonly noted in all the control rats. In particular, prominent intra-alveolar hemorrhage occurred in those challenged with risperidone, amphetamine, and combined amphetamine and haloperidol. Contrarily, in the rats treated with BPC 157, these lesions were markedly attenuated (only mild congestion) or even completely annihilated in the rats challenged with fluphenazine and combined amphetamine and haloperidol.

#### 2.2.4. Liver

Considerable dilatation and congestion of blood vessels in the portal tracts, central veins, and sinusoids were found in all the control rats, in particular in those challenged with fluphenazine, risperidone, olanzapine, quetiapine, aripiprazole, and combined amphetamine and haloperidol. Contrarily, in the rats treated with BPC 157, these lesions were markedly attenuated (only mild dilatation and congestion) or even completely annihilated in the rats challenged with haloperidol, aripiprazole, and amphetamine.

#### 2.2.5. Kidney

Considerable dilatation and congestion in the tissue, as well as glomeruli, occurred in all the control rats, in particular in those challenged with quetiapine, aripiprazole, domperidone, and amphetamine. Contrarily, in the rats treated with BPC 157, these lesions were markedly attenuated (only mild dilatation and congestion) or even completely annihilated in the rats challenged with haloperidol, aripiprazole, and amphetamine.

#### 2.2.6. Gastrointestinal Lesions

**Figure 15:**
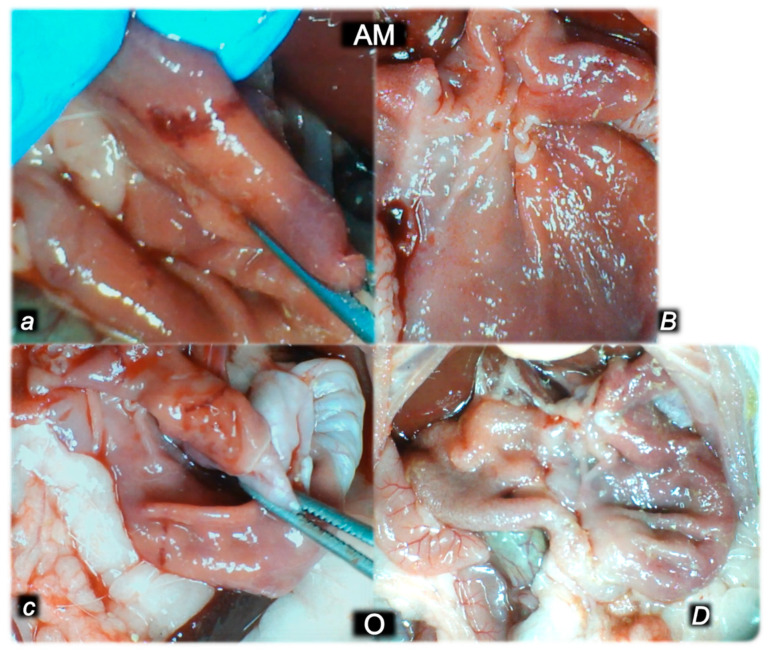
Illustrative presentation of stomach lesions in the control rats (*italic small letters*) and lack of stomach lesions in the BPC 157-treated rats (*italic capital letters*) at 15 min following application of amphetamine (AM) (*a,B*) or olanzapine (O) (*c,D*).

Marked stomach hemorrhagic lesions, gross (Figure 15) and microscopic, and considerable congestion of the stomach wall occurred in all the control rats, in particular in those challenged with combined amphetamine and haloperidol. Contrarily, in the rats treated with BPC 157, there were no gross lesions nor microscopic congestion of the stomach wall.

### 2.3. A Perilous Syndrome Occurred Centrally

#### Brain Lesions, Cerebral and Cerebellar Cortex, Hypothalamus/Thalamus, and Hippocampus

**Figure 16:**
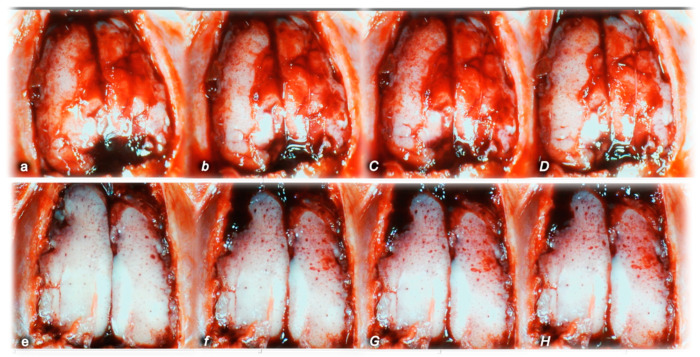
Brain presentation before the challenge, after the challenge (haloperidol or amphetamine) application, and after the therapy application. Normal brain presentation (normal small letters) and brain swelling (*italic small letters*) upon noxious agent (haloperidol (upper) or amphetamine (lower)) application (*italic small letters*) and counteracted brain swelling after BPC 157 application (*italic capital letters*). Upper. Brain presentation in the normal healthy rat (a), brain swelling presentation immediately upon haloperidol application (*b*), decreased brain swelling immediately upon BPC 157 administration (*C*), and decreased brain swelling in BPC 157 treated rat immediately before sacrifice (*D*). Lower. Brain presentation in the normal healthy rat (e), brain swelling presentation immediately upon amphetamine application (*f*), decreased brain swelling immediately upon BPC 157 administration (*G*), and decreased brain swelling in BPC 157 treated rat immediately before sacrifice (*H*). A similar presentation occurred also with other dopamine agents’ applications and BPC 157 therapy.

We assumed a failed common clue (i.e., intracranial (superior sagittal sinus), portal, and caval hypertension, aortal hypotension, progressed thrombosis, peripherally and centrally, failed collateral recruitment, disturbed ECG presentation, peripheral organ lesions). Namely, all the dopamine agents (haloperidol, fluphenazine, clozapine, risperidone, olanzapine, quetiapine, aripiprazole, domperidone, amphetamine, and amphetamine combined with haloperidol) converge to the similar brain lesion as well. Already in the immediate post-application period, there was increased intracranial (superior sagittal sinus) hypertension, along with the brain being consistently swollen, severe brain edema and congestion, and prominent intracerebral cortical hemorrhage. Contrarily, BPC 157 therapy reduced intracranial (superior sagittal sinus) hypertension (and eliminated portal and caval hypertension), along with counteracted brain swelling (Figure 16), markedly counteracted brain edema and congestion, and counteracted hemorrhage. Furthermore, the rats challenged with amphetamine as well as the rats challenged with haloperidol were similarly cured with BPC 157 therapy applied intragastrically and BPC 157 therapy applied intraperitoneally, providing beneficial effects that might be equally achieved by either regimen (data not specifically shown).

Pronounced edema and congestion were visible in the brain tissue in all control rats according to the assessed brain areas (Table 5). In addition, a more prominent intracerebral cortical hemorrhage involving larger areas of brain tissue occurred, affecting areas of the corpus callosum (haloperidol, domperidone, amphetamine, and combined amphetamine and haloperidol) and neocortex (haloperidol, fluphenazine, olanzapine, domperidone, quetiapine, aripiprazole, amphetamine, and combined amphetamine and haloperidol). In contrast, only mild edema and congestion occurred in the BPC 157 groups, with small, focal, and superficial areas of neocortical hemorrhage in haloperidol, fluphenazine, olanzapine, quetiapine, domperidone, amphetamine, and combined amphetamine and haloperidol challenged rats.

In addition, all control rats presented severe neurodegenerative changes in the central nervous system. We noted an increased number of karyopyknotic cells markedly affecting all four regions: cerebral and cerebellar cortex, hypothalamus/thalamus, and hippocampus. There was karyopyknosis and degeneration of cortical neurons and pyramidal cells of the hippocampus, followed by karyopyknosis and degeneration of Purkinje cells of the cerebellar cortex. These were all attenuated in BPC-157-treated rats.

### 2.4. Perilous Syndrome with Concomitant Application of the Dopamine Antagonist (Haloperidol) and Dopamine Agonist (Amphetamine)

**Figure 17:**
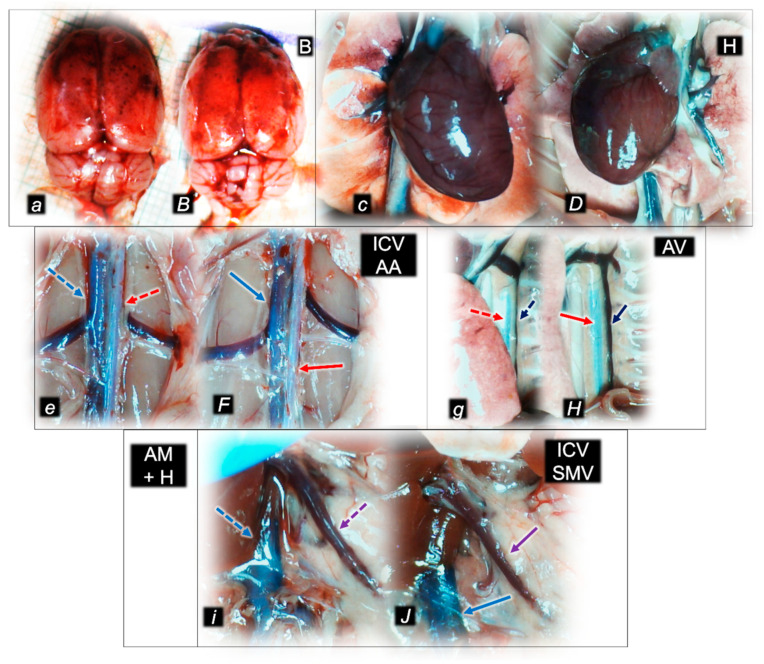
Illustrative presentation of the unusual parallel activity of the haloperidol and amphetamine in the amphetamine and haloperidol combined challenged rats (AM+H), controls (*italic small letters*, dashed arrows) and BPC 157 treated (*italic capital letters*, full arrows) in the brain (B) (*a, B*), heart (H) (*c, D*), inferior caval vein (blue arrows) and abdominal aorta (red arrows) (ICVAA) (*e, F*), azygos vein (blue arrows) (AV) (*g, H*), and superior mesenteric vein (violet arrows) and inferior caval vein (blue arrows) (ICVSMV) (*i, J*). These changes occurred probably beyond the dopamine system since given together haloperidol and amphetamine did not antagonize each other effect, and the complete syndrome remained. However, it was consistently antagonized by BPC 157 application.

**Figure 18:**
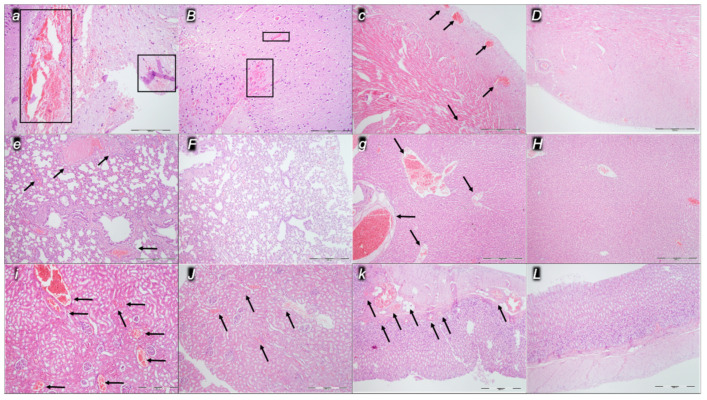
Amphetamine and haloperidol-rats (amphetamine+haloperidol). Illustrative microscopic presentation of the brain (*a*, *B*), heart (*c*, *D*), lung (*e*, *F*), liver (*g*, *H*), kidney (*i*, *J*), and stomach (*k*, *L*) in the saline-treated rats (control, *italic small letters*) and BPC 157-treated rats (*italic capital letters*) at the end of the experiments at the 15 min. Brain (*a*, *B*). Control (*a*). A pronounced edema and congestion were visible affecting the cerebrum, and more prominent intracerebral cortical hemorrhage involving larger areas of cerebral brain tissue affecting the periventricular area (rectangular areas). BPC 157 (*B*). Only mild edema and congestion were found, with small, focal, and superficial areas of periventricular hemorrhage (rectangular areas). Heart (*c*, *D*). Control (*c*). Marked myocardial congestion (black arrows). BPC 157 (*D*). No changes were found. Lung (*e*, *F*). Control (*e*). A marked congestion of the lung parenchyma with intra-alveolar hemorrhage (black arrows). BPC 157 (*F*). No changes were found. Liver (*g*, *H*). Control (*g*). A marked dilatation and congestion of blood vessels in the portal tracts, central veins, and sinusoids (black arrows). BPC 157 (*H*). No changes were found. Kidney (*i*, *J*). Control (*i*). A moderate dilatation of blood vessels and congestion in the kidney tissue, as well as glomeruli, was found (black arrows). BPC 157 (*J*). Mild dilatation of blood vessels and congestion in the kidney tissue, as well as glomeruli (black arrows). Gastrointestinal lesion (*k*, *L*). Control (*k*). A marked congestion of the stomach wall (black arrows). BPC 157 (*L*). No changes were found in the stomach wall. HE; magnification 100× (*c*, *D*, *e*, *F*, *g*, *H*, *i*, *J*, *k*, *L*), magnification 200× (*a*, *B*).

Application of haloperidol, fluphenazine, clozapine, risperidone, olanzapine, quetiapine, aripiprazole, domperidone, amphetamine, and combined amphetamine and haloperidol all produced similar portal and caval hypertension, aortal hypotension, superior sagittal sinus hypertension, progressed thrombosis, organ lesions, and considerable but distinctive ECG disturbances (i.e., prolonged QTc intervals (antipsychotics, domperidone), short QTc intervals (amphetamine)). Thereby, an unusual parallel activity of haloperidol and amphetamine (providing opposite effects of dopamine antagonists and dopamine agonists) occurred, probably beyond the dopamine system since, generally, given together, haloperidol and amphetamine did not antagonize each other, and the complete syndrome remained (Figure 17 and Figure 18). However, in particular with ECG disturbances, some mutual antagonization might occur; prolonged PQ intervals and opposite QTc intervals might antagonize each other (while tachycardia remained) (Table 3). However, these were all consistently antagonized by BPC 157 application.

In summary, after BPC 157 therapy, the occlusion/occlusion-like syndrome course was markedly attenuated/eliminated in all rats. These occurred with haloperidol, fluphenazine, clozapine, risperidone, olanzapine, quetiapine, aripiprazole, domperidone, amphetamine, and amphetamine and haloperidol combined. These rats exhibited no portal and caval hypertension, ameliorated aortal hypotension, markedly attenuated superior sagittal sinus hypertension, attenuated tachycardias, and had not changed PQ interval or QTc interval. Additionally, venous and arterial thrombosis was attenuated, both peripherally and centrally, reduction of the brain and internal organs lesions in the heart, lung, liver, kidney, and gastrointestinal tract. Thus, BPC 157 therapy, given in either of regimens (µg, ng, ip, ig) counteracted the adverse effects that would otherwise consistently appear along with the large range of the distinctive dopamine agonists, dopamine antagonists, central (antipsychotics), peripheral (prokinetic, domperidone), and agonist (amphetamine). Note, there is the uniformity of these adverse effects obtained with distinctive dopamine agents application, dopamine antagonists, central (antipsychotics), peripheral (prokinetic, domperidone), and agonist (amphetamine). Likewise, there is evidence that these shared disturbances sustainably appeared even with the combination of amphetamine and haloperidol (agonist and antagonist do not inhibit each other effect). This suggests that the consistent antagonization exerted by the BPC 157 therapy probably takes place also beyond the dopamine system. The key finding of an activated particular collateral pathway, i.e., the azygos vein, which combined the inferior caval vein and left superior vein to reorganize blood flow, might be responsible for the noted beneficial effects.

## 3. Discussion

The important issue of brain-gut and gut-brain axes (mal)functioning [1] is in general focus throughout the research community. As a new practical point, we revealed the full new topic and essential matching of central and peripheral occlusion/occlusion-like syndrome with haloperidol, fluphenazine, clozapine, olanzapine, risperidone, quetiapine, aripiprazole, domperidone, amphetamine, and combined amphetamine and haloperidol, and all particularities. These shared disturbances were consistently reversed by the stable gastric pentadecapeptide BPC 157 (i.e., activated azygos vein, direct blood flow delivery to re-establish reorganized blood flow) [1]. There, the consistent disturbance, as the effects of a multitude of agents, might be overwhelmed by the consistent effects of BPC 157 regimens (µg-ng, intraperitoneal, intragastric, given at 5 min after dopamine agents). Thus, the consistent disturbances, and the consistent therapy effects, both support each other effect (i.e., probably also in relationships that they are supposed to mimic (i.e., catalepsy, schizophrenia symptoms [28,29])). The whole disturbance was analogous to the occlusion/occlusion-like syndrome in rats with permanent occlusion of the major vessel(s) peripherally [14,15,16] or centrally [18] and similar noxious procedures [20,21,22,23,24] severely damaging endothelial function. Likewise, BPC 157 therapy effect was the same resolving multicausal pathology. There, in rats, which were damaged, several important aspects of rapidly progressing disturbances should be further analyzed. These imply brain lesions (widespread hemorrhage), heart (congestion and infarctions), lung (hemorrhage), congestion in the liver, kidney, and gastrointestinal tract, arrhythmias, and intracranial (superior sagittal sinus), portal and caval hypertension, and aortal hypotension. Disabled were major vessels (i.e., congested inferior caval vein and superior mesenteric vein, collapsed azygos vein). Arterial and venous thrombosis were peripherally and centrally, and widespread Virchow triad circumstances were generally presented. 

In the timeline, the instant onset of the occlusion/occlusion-like syndrome (and the BPC 157 therapy effect as well) preceded the period where the behavioral disturbances may appear. This may be important for the given evidence that BPC 157 therapy also counteracted the neuroleptic- or L-NAME-induced catalepsy, and the schizophrenia positive and negative symptoms (amphetamine, apomorphine, methamphetamine, ketamine) [25,26,27,28,29]. Thus, we can argue with BPC 157 as a prototype cytoprotective agent [1,2,3,4,5,6,7] (i.e., activation of collateral (azygos vein) pathway as an outbreak of the endothelium protection long ago recognized as an innate effect of cytoprotective agents) novel common therapy solution within the new cytoprotection issue. This may imply the various typical and atypical neuroleptics-, amphetamine-, prokinetic domperidone-induced disturbances, acting centrally and/or peripherally, thus disturbances in general. Presenting the agents’ supposed major activity (central (neuroleptics) [55,56,57], peripheral (domperidone) [58], peripheral and central (amphetamine) [59]) this may be whatever the occlusion/occlusion-like syndrome occurred from centrally (i.e., neuroleptics), or from the periphery (i.e., domperidone) or simultaneously at the peripheral and central sides (i.e., amphetamine). Note, rats with the occluded superior mesenteric vein, occluded superior mesenteric artery, or both artery and vein, immediately presented intracranial (superior sagittal) hypertension in addition to portal and caval hypertension and aortal hypotension [14,15,16]. Likewise, rats with occluded superior sagittal sinus, in addition to intracranial (superior sagittal sinus) hypertension immediately presented also portal and caval hypertension and aortal hypotension [18]. Consistently, in these experiments, BPC 157 might equally act given centrally topically (i.e., at the brain) or given peripherally, intraperitoneally, or intragastrically [18]. This might be three body cavities interconnected and rapidly communicating with each other, and disturbances rapidly transmitted through the venous system, unless rapidly upgraded by the given therapy [14,15,16,18,20,21,22,23,24]. This was especially evidenced in rats with permanent mechanically maintained severe intra-abdominal hypertension, grade III and IV. Receiving BPC 157 therapy provided that rats smoothly sustained intra-abdominal hypertension, even grade III and IV, without major harm [21]. 

General important points are long-ago described agenda for gut peptides in brain-gut and gut-brain axes, malfunctioning and functioning, based on the evidenced peripheral and central effects on the internal organs and brain lesions [1]. Thereby, neuroleptics, domperidone, and amphetamine effects presented a common periphery-central and central-periphery link and a common model throughout the occlusion/occlusion-like syndrome of tightly interconnected vascular and multiorgan failure. Corresponding multicausal pathology was in occlusion/occlusion-like syndromes in rats with permanent occlusion of the major vessel(s) peripherally [14,15,16] or centrally [18] and similar noxious procedures [20,21,22,23,24] severely damaging endothelial function. Thereby, the general significance and the beneficial role of the stable gastric pentadecapeptide BPC 157, maintaining epithelium and endothelium integrity is the strong counteracting potential of activation of the collaterals, i.e., azygos vein direct blood flow delivery to the attenuated/eliminated occlusion/occlusion-like syndrome, pleiotropic beneficial effects to reestablish the needed brain-gut and gut-brain axes functioning [1,2,3,4,5,6,7].

Specifically, this report of occlusion/occlusion-like syndrome emphasized the essential matching between the prototypic neuroleptics, haloperidol, fluphenazine, clozapine, olanzapine, risperidone, quetiapine, and aripiprazole. These common points may confirm the occlusion/occlusion-like syndrome to be a class-adverse effect of neuroleptics. Likewise, there is an essentially equal occlusion/occlusion-like syndrome after domperidone. Finally, the outcome of amphetamine had the same occlusion/occlusion-like syndrome. Thus, in addition to the quite distinctive medical application of these agents (i.e., psychosis, attention deficit hyperactivity disorder (ADHD), narcolepsy, nausea, and vomiting) [55,56,57,58,59], we claimed the common neuroleptic–amphetamine–domperidone occlusion/occlusion-like syndrome as a shared class-adverse effect of dopamine agents from distinctive classes. As a supporting fact, this huge overlapping occlusion/occlusion-like syndrome as a particular commonality overwhelms or combines seemingly opposite circumstances. Blocked were various receptors. Dopamine D2 receptor antagonists haloperidol and fluphenazine [55,56] blocked muscarinic, adrenergic, histamine, serotonin, and dopamine receptors. D4 receptors and 5-HT2A receptors blockade was with clozapine, risperidone, olanzapine, and aripiprazole [55,56]. Quetiapine blocked mostly 5-HT-2A [57]. Domperidone [58] blocked peripheral D2 and D3 receptors. Amphetamine increased the neurotransmission of dopamine, serotonin, adrenaline, noradrenaline, and histamine [60,61]. From a therapy point, this requires also a special combining effect [1]. 

Furthermore, as it was in rats with major vessel occlusion or who underwent similar noxious procedures [14,15,16,18,20,21,22,23,24], there are multiple improved outcomes in each organ. These evidenced that BPC 157 beneficial effects can be promptly shared in all of the neuroleptics-, amphetamine- and domperidone-rats as common resolution with BPC 157 therapy. Commonly, there was rapid counteraction of the brain swelling (gross assessment), brain lesions and intracerebral hemorrhage (microscopy), and intracranial (superior sagittal sinus) hypertension counteracted. Thus, there was rapidly restored essential ability to drain venous blood adequately for a given cerebral blood inflow without rising venous pressures [14,15,16,18,20,21,22,23,24]. Also, counteraction of the heart dilatation and myocardial congestion occurred as a common point, counteraction of prolonged PQ-intervals, and prolonged QTc-interval (neuroleptics, domperidone) and short QTc-interval (amphetamine) in particular toward the given agent, all arrhythmias in general. Note, considering the particular anti-arrhythmic potential of BPC 157 therapy [4,5], long QT occurred with neuroleptics [62,63,64,65,66,67]. Shortened QT interval [67,68,69,70,71] occurring with increased levels of catecholamine (amphetamine) can degenerate into paroxysmal atrial fibrillation and/or ventricular tachycardia, potentially leading to sudden cardiac death [67,68,69,70,71,72]. Further, along with annihilated thrombosis, peripherally and centrally, heart failure recovered as a whole [4,5] always appeared as a common effect. Lungs, liver, kidney, and gastrointestinal tract lesions and intra-alveolar hemorrhage in the lungs markedly attenuated or even eliminated, may clearly reflect biventricular heart failure fully reversed with BPC 157 therapy. As the initial cause-consequence chain of events, the congested inferior caval vein and superior mesenteric vein became decongested. Initially collapsed azygos vein was instantly recovered as a rescuing pathway (direct blood flow delivery). Thus, the reorganized blood flow was rapidly re-established. Eliminated were the otherwise severe caval and portal hypertension and aortal hypotension, eliminated were Virchow triad circumstances in general. Note, as part of the counteraction of Virchow triad circumstances in general, counteracted were both progressed thrombosis and widespread hemorrhage (i.e., brain, lung) as it was the combined counteraction in other occlusion/occlusion-like syndromes [14,15,16,18,20,21,22,23,24].

Thereby, BPC 157 in its role in brain-gut and gut-brain axes functioning [1] may recover neuroleptic-domperidone-amphetamine-occlusion/occlusion-like syndrome by competing with all receptor blockade (neuroleptics, domperidone) [55,56,57,58]. This should be along with the counteraction of the increased neurotransmission of dopamine, serotonin, adrenaline, noradrenaline, and histamine (amphetamine) [59,60,61] (note, BPC 157 therapy was at 5 min after agents’ application). Alternatively, it may be that BPC 157 may fully exert its beneficial effect and avoid the consequences through an innate cytoprotective activity and vascular recovery [1,2,3,4,5,6,7] while receptor blockade and increased neurotransmission of dopamine, serotonin, adrenaline, noradrenaline, and histamine [55,56,57,58,59,60,61] might remain unchanged but harmless. Note, such essential cytoprotection background was suggested, as BPC 157 therapy was fully effective during the permanent occlusion of major vessel, peripheral [14,15,16] and central [18], or mechanically maintained grade III and grade IV of intra-abdominal hypertension and compressed vessels and organs [21]. With BPC 157 therapy, in particular, this is also the rapid upgrading of the minor vessel to activate rescuing collateral pathways to take over and compensate for failed major vessel dysfunction and reestablish the reorganized blood flow [14,15,16,18,20,21,22,23,24]. Indeed, there were molecular changes, rapidly changing lipid contents and protein secondary structure conformations in rat vessels produced instantly by BPC 157 therapy (Fourier-transform infrared spectroscopy), lack of cell death, and consequently, the increased capability of the rat vessel (i.e., thoracic aorta) to function even in the worst circumstances [73]. Thus, since long ago, cytoprotection activity was related to the rapidly improved innate endothelium maintenance [30,31,32,33,34,35], there is an innate response that might ascertain particular action [1,2,3,4,5,6,7]. To resolve neuroleptic-amphetamine-domperidone-occlusion/occlusion-like syndrome, it should accordingly deal equally with the neuroleptics-central receptors blockade [55,56,57], domperidone-peripheral receptors blockade [62] as well as amphetamine-increased neurotransmission [59,60,61]. 

Finally, it might be that unable to oppose each other’s full harmful effects, combined amphetamine (agonist) and haloperidol (antagonist) together (amphetamine+haloperidol) perpetuated the injurious circle likely within described injury-specific cytoprotection background not specifically related to any receptor disability [1,2,3,4,5,6,7]. However, such a shared class effect may pick up the particular dopamine points that were mutually antagonized, ECG-disturbances (prolonged PQ-intervals, and prolonged QTc-interval (haloperidol) and short QTc-interval (amphetamine) were mutually normalized). These may be special targets, specifically the dopamine system related. On the other hand, the whole perpetuated injurious circle was completely opposed by the stable gastric pentadecapeptide BPC 157 therapy; the BPC 157-related background might be essential.

Summarizing, once presented, the neuroleptic-amphetamine-domperidone-occlusion/occlusion-like syndrome as the indicative complex outcome may precede, or even specifically cause dopamine antagonists- and dopamine agonists-induced harmful effects. Likely, this may be interplaying with the dysfunction of other systems known to be essential in the cytoprotection concept and central lesion development [1,9,10], such as NO-system [50,51,52,53,54,74,75]. Contrarily, BPC 157 induces the NO release on its own [52,53]) and prostaglandins-system maintenance (i.e., BPC 157 counteracted NSAIDs-induced central and peripheral lesions and bleeding disorders [76,77,78,79]). Indicatively, as with the dopamine system, a particular controlling system (i.e., activation of the suited collateral pathways) might be the modulatory effectiveness depending on the injury state. Illustrative may be the consistent counteraction of the adverse effects of NO-synthase (NOS) blocker (L-NAME-induced hypertension and pro-thrombotic effect [52,54]), and NOS substrate (L-arginine-induced hypotension and anti-thrombotic effect [52,54]). With BPC 157 therapy, the VEGFR2-Akt-eNOS signaling pathway is activated without the need for other known ligands or shear stress [75], and the vasomotor tone, both smooth muscle and endothelium, maintained through the activation of Src-Caveolin-1-eNOS pathway [74]. Besides, BPC 157 might specifically maintain the function of thrombocytes without affecting coagulation pathways [54,78,79]. Moreover, BPC 157 may have the additional possible controlling capacity, equally effective given in the ischemia [14,15,16,18] as well as after initiated reperfusion centrally (stroke [19], spinal cord compression [80,81]) or peripherally (Pringle maneuver, ischemic/reperfusion colitis) [12,82]. There is interaction with many molecular pathways [11,19,74,75,77,83,84,85,86,87,88,89,90] (i.e., BPC 157 counteracted tumor-induced cachexia [90]), and counteracting pro-inflammatory and pro-cachectic cytokines pathways. In particular, in wound healing [8,91], it may at the same time increase both the early growth response protein 1 (egr1)-gene and its corepressor gene NGFI-A-binding protein 2 (naB2) expression [86]. In addition, BPC 157 counteracted leaky gut as a stabilizer of cellular junctions [87], and acts as a free radical scavenger [92,93,94,95], in particular in vascular occlusion studies [11,12,13,14,15,16,20,22]. 

## 4. Materials and Methods

### 4.1. Animals

Male Albino Wistar rats, 12 weeks old, 200 g body weight, bred in-house at the Animal Pharmacology Facility, School of Medicine, Zagreb, Croatia (registered with the Veterinary Directorate (Reg. No: HR-POK-007)), randomly assigned at 6 rats/group/interval, were used in all experiments. Rats were acclimated for five days and randomly assigned to their respective treatment groups; they were housed in polycarbonate (PC) cages (identified with dates, number of study, group, dose, number, and sex of each animal) at 20–24 °C, relative humidity of 40–70% and noise level 60 dB, illumination 12 h per day (fluorescent lighting), standard good laboratory practice (GLP) diet and fresh water ad libitum. Procedures were in accordance with the standard operating procedures (SOPs) of the Animal Pharmacology Facility and the European Convention for the Protection of Vertebrate Animals used for Experimental and other Scientific Purposes (ETS 123). This study was approved by the local Ethics Committee. Ethical principles of the study complied with the European Directive 010/63/E, the Law on Amendments to the Animal Protection Act (Official Gazette 37/13), the Animal Protection Act (Official Gazette 135/06), the Ordinance on the protection of animals used for scientific purposes (Official Gazette 55/13), Federation of European Laboratory Animal Science Associations (FELASA) recommendations, and the recommendations of the Ethics Committee of the School of Medicine, University of Zagreb. The experiments were assessed by observers blinded to the treatment.

### 4.2. Drugs

Stable gastric pentadecapeptide BPC 157 (GEPPPGKPADDAGLV, molecular weight 1419; Diagen, Slovenia), a partial sequence of the human gastric juice protein BPC, which is freely soluble in water at pH 7.0 and in saline, was prepared as a peptide with 99% high-performance liquid chromatography (HPLC) purity, with 1-des-Gly peptide being the main impurity [1,2,3,4,5,6,7,8,9,10]. Haloperidol, fluphenazine, clozapine, risperidone, olanzapine, quetiapine, aripiprazole, domperidone, and amphetamine were commercially purchased (Sigma, Aldrich, St. Louis, MO, USA).

### 4.3. Experimental Protocol

In deeply anesthetized rats (40 mg/kg thiopental (Rotexmedica, Germany) and 10 mg/kg diazepam (Apaurin; Krka, Slovenia) intraperitoneally), complete calvariectomy was performed; to induce rapid vascular failure and concomitant general syndrome, we applied (mg/kg) intraperitoneally haloperidol (5), fluphenazine (5), clozapine (10), risperidone (5), olanzapine (10), quetiapine (10), or aripiprazole (10), domperidone (25), amphetamine (10), and combined amphetamine and haloperidol, with an assessment at 15 min.

Rats received as therapy BPC 157 (10 µg or 10 ng/kg) or saline (5 mL/kg) (controls) as an early intraperitoneal regimen at 5 min upon haloperidol, fluphenazine, clozapine, risperidone, olanzapine, quetiapine, aripiprazole, domperidone, amphetamine, and combined amphetamine and haloperidol. As an early regimen, rats received BPC 157 or saline (5 mL/kg) as an intragastric administration at 5 min following haloperidol or amphetamine.

After complete calvariectomy, recordings of brain swelling (before the procedure, after haloperidol, fluphenazine, clozapine, risperidone, olanzapine, quetiapine, or aripiprazole, domperidone, amphetamine, and combined amphetamine and haloperidol, after therapy application, and before sacrifice) followed the procedure previously used in our vascular studies, with permanent major vessel occlusion peripheral [14,15,16] and central [18], and similar procedures application [20,21,22,23,24]. Calvariectomy procedure included, medially to the superior temporal lines and temporalis muscle attachments, 6 burr holes drilled in three horizontal lines (just basal from the posterior interocular line (two rostral burr holes); just rostral to the lambdoid suture (and transverse sinuses) on both sides (two basal burr holes); in line between the basal and rostral burr holes (two middle burr holes)).

Rats were laparatomized again before sacrifice for the corresponding presentation of the peripheral vessels (azygos vein, superior mesenteric vein, portal vein, inferior caval vein) and corresponding organ lesions (i.e., stomach lesion). The recording was performed with a camera attached to a VMS-004 Discovery Deluxe USB microscope (Veho, Claymont, DE, USA) at the end of the experiment, and assessed as before [14,15,16,18,20,21,22,23,24].

### 4.4. Superior Sagittal Sinus, Portal, Caval Vein, and Abdominal Aorta Pressure Recording

Recordings followed the procedure used and described in detail in our previous vascular studies with permanent major vessel occlusion peripheral [14,15,16] and central [18], and similar procedure application [20,21,22,23,24] (deeply anesthetized rats, a cannula (BD Neoflon™ Cannula) connected to a pressure transducer (78534C MONITOR/TERMINAL; Hewlett Packard, Palo Alto, CA, USA), inserted into the portal vein, inferior caval vein, and superior sagittal sinus, as well as the abdominal aorta at the level of the bifurcation at 15 min after haloperidol, fluphenazine, clozapine, risperidone, olanzapine, quetiapine, or aripiprazole, domperidone, and amphetamine, and combined amphetamine and haloperidol. The superior sagittal sinus anterior part was cannulated using a Braun intravenous cannula, and then, after laparotomy, the pressure in the portal vein, inferior vena cava, and abdominal aorta was recorded.

Accordingly [14,15,16,18,20,21,22,23,24], superior sagittal sinus pressure of −24 to −27 mm Hg, portal pressure of 3–5 mm Hg similar to that of the inferior vena cava, although with values at least 1 mm Hg higher in the portal vein, and abdominal aorta blood pressure values 100–120 mm Hg at the level of the bifurcation were considered to be normal in healthy rats.

### 4.5. ECG Recording

ECGs were recorded continuously in deeply anesthetized rats for all three main leads, by positioning stainless-steel electrodes on all four limbs using an ECG monitor with a 2090 programmer (Medtronic, Minneapolis, MN, USA) connected to a Waverunner LT342 digital oscilloscope (LeCroy, Chestnut Ridge, NY, USA) (before the procedure, after haloperidol, fluphenazine, clozapine, risperidone, olanzapine, quetiapine, or aripiprazole, domperidone, amphetamine, and combined amphetamine and haloperidol, after therapy application, and before sacrifice). This arrangement enabled precise recordings, measurements, and analysis of ECG parameters (PQ intervals, QTc, heart frequency) as specifically described [14,15,16,18,20,21,22,23,24].

### 4.6. Thrombus Assessment

Following sacrifice, the superior sagittal sinus and, peripherally, the portal vein, inferior caval vein, and abdominal aorta were removed from the rats, and the clots were weighed (Sartorius analytic A 200 S) [14,15,16,18,20,21,22,23,24].

### 4.7. Brain Volume, Heart, and Vessel Volume Presentation

The applied procedure was used in our previous vascular studies [14,15,16,18,20,21,22,23,24]. Brain volume, vessel volume, and heart volume were proportional to the change in the brain or vessel or heart surface area. The presentation of the brain and peripheral vessels (superior mesenteric vein, inferior caval vein, azygos vein, and abdominal aorta) was recorded in deeply anesthetized rats, with a camera attached to a VMS-004 Discovery Deluxe USB microscope (Veho, Claymont, DE, USA). The border of the brain (or vessels or heart) in the image was marked using ImageJ software, and then the surface area of the brain (or vessels or heart) was measured. This was done with the brain (or vein) images for healthy rats, and then for both the control (saline) group and treated (BPC 157) group of rats at the same intervals after the application and at the time of sacrifice. The arithmetic mean of the surface areas was calculated for both groups. Then, the ratio of these two areas was calculated as ($\frac{A_{con}}{A_{bpc}}$), where Acon is the arithmetic mean brain (or veins or heart) area of the control group, and Abpc is the arithmetic mean brain (or veins or heart) area of the treated group. Starting from the square-cube law, Equations (1) and (2), an equation for the change in brain (or veins or heart) volume proportional to the change in brain surface area (or veins, or heart) (6), were derived. In expressions (1)–(5), *l* is defined as any arbitrary one-dimensional length of the brain (for example, rostro-caudal length of the brain) (or veins or heart), used only for defining the one-dimensional proportion (*l*_2_/*l*_1_) between two observed brains (or veins) and as an inter-factor (and because of that not measured (6)) for deriving final expression (6). The procedure was as follows:

(1) $$A_{2}=A_{1}\times {\left(\frac{l_{2}}{l_{1}}\right)}^{2}$$

(square-cube law),

(2) $$V_{2}=V_{1}\times {\left(\frac{l_{2}}{l_{1}}\right)}^{3}$$

(square-cube law),

(3) $$\frac{A_{2}}{A_{1}}={\left(\frac{l_{2}}{l_{1}}\right)}^{2}$$

(from (1), after dividing both sides by A1),

(4) $$\frac{l_{2}}{l_{1}}=\sqrt{\frac{A_{2}}{A_{1}}}$$

(from (3), after taking the square root of both sides),

(5) $$\frac{V_{2}}{V_{1}}={\left(\frac{l_{2}}{l_{1}}\right)}^{3}$$

(from (2), after dividing both sides by V1),

(6) $$\frac{V_{2}}{V_{1}}={\left(\sqrt{\frac{A_{2}}{A_{1}}}\right)}^{3}$$

(after incorporating expression (4) into Equation (5)).

### 4.8. Gross Assessment of Gastrointestinal Lesions

For recording, we used a camera attached to a VMS-004 Discovery Deluxe USB microscope (Veho, Claymont, DE, USA). As described before, gross lesions in the gastrointestinal tract—the stomach, in particular—(sum of the longest diameters, mm) were assessed in deeply anesthetized rats, laparatomized before sacrifice [14,15,16,18,20,21,22,23,24].

### 4.9. Microscopy

As described in the previous studies [14,15,16,18,20,21,22,23,24], evaluation was done by light microscopy using an Olympus 71 digital camera and an Olympus BX51 microscope (OLYMPUS Europa SE & Co. KG). Digital images were saved as uncompressed 24-bit RGB TIFF files using the software program AnalySIS (Olympus Soft Imaging System GmbH, Munster, Germany). Representative tissue specimens (i.e., the brain, liver, kidney, stomach, small and large intestine, lungs, and heart taken at the end of the experiment, fixed in 10% neutral buffered formalin (pH 7.4) at room temperature for 24 h) were embedded in paraffin, sectioned at 4 μm, and stained with hemalaun and eosin (H&E).

#### 4.9.1. Brain Histology

As described in the previous studies [14,15,16,18,20,21,22,23,24], the brain was dissected according to NTP-7 at Level 3 and 6 with neuroanatomic subsites presented in certain brain sections using coronal sections with three mandatory sections. We used a semiquantitative neuropathological scoring system, and the sum of analyzed affected areas (0–4) (i) and karyopyknotic cells in the brain areas (0–4) (ii) making (i) + (ii) a combined score (0–8), as follows. (i). Specifically affected brain areas (cerebral (NTP-7, Level 3)—cerebellar cortex (NTP-7, Level 6), hippocampus, thalamus, and hypothalamus (NTP-7, Level 3))—were scored (0–4) (score 0 indicates no histopathologic change), as follows. Small, patchy, complete, or incomplete infarcts (≤10% of area affected) represented score 1. Partly confluent or incomplete infarcts (20–30% of area affected) represented score 2. Large confluent complete infarcts (40–60% of area affected) represented score 3. In the cortex total disintegration of the tissue, and in the hypothalamus, thalamus, and hippocampus large complete infarcts (˃75% of the area affected) represented score 4. (ii) Analysis was conducted on karyopyknotic cells in the affected brain areas (0–4) (score 0 indicates no change), cerebral (NTP-7, Level 3), cerebellar cortex (NTP-7, Level 6), hippocampus, thalamus, and hypothalamus (NTP-7, Level 3) as follows: a few karyopyknotic of neuronal cells (≤20%) (score 1); patchy areas of karyopyknotic cells (50%) (score 2); more extensive karyopyknotic areas (75%) (score 3); complete infarction (100%) (score 4). Brain tissue hemorrhage was obtained by estimating a percentage of the affected areas. Intraventricular hemorrhage was noted as present or absent.

We also assessed the neuronal pathological changes in acquired digital images saved as uncompressed 24-bit RGB TIFF files in the software program AnalySIS (Olympus Soft Imaging System GmbH, Munster, Germany) performing quantitative analysis of neuronal damage in the karyopyknotic areas. The neurons of the cortical cerebral, cerebellar region, hippocampus, and hypothalamus were counted in 10 different high-powered fields (HPF, 400×), and 3 to 5 serial sections of each sample were used to conduct the count as described [96]. The field size was 0.24 μm^2^.

We used four criteria for the estimation of the edema: pale myelin, sieve-like appearance of myelinated areas, dilation of perivascular and pericellular spaces, and vacuolar appearance of the neuropil of gray matter. Edema was graded as heavy, moderate, slight, or no edema (score 0–3) [97].

#### 4.9.2. Lung Histology

The same scoring system as in the previous studies [14,15,16,18,20,21,22,23,24] was used to grade the degree of lung injury in lung tissue analysis. Each of the features (i.e., focal thickening of the alveolar membranes, congestion, pulmonary edema, intra-alveolar hemorrhage, interstitial neutrophil infiltration, and intra-alveolar neutrophil infiltration) was scored (0–3) as absent (0) or present to a mild (1), moderate (2), or severe (3) degree, and a final histology score was determined.

#### 4.9.3. Renal, Liver, and Heart Histology

The same scoring system as in the previous studies [14,15,16,18,20,21,22,23,24] was used to grade renal (i.e., the degeneration of Bowman’s space and glomeruli, degeneration of the proximal and distal tubules, vascular congestion, and interstitial edema), liver (i.e., vacuolization of hepatocytes and pyknotic hepatocyte nuclei, activation of Kupffer cells, and enlargement of sinusoids), and heart (i.e., dilatation and congestion of blood vessels within the myocardium and coronary arteries) histology. Each specimen was scored using a scale ranging from 0 to 3 (0: none, 1: mild, 2: moderate, and 3: severe) for each criterion, and a final histology score was determined (0: none, 1: mild, 2: moderate, and 3: severe).

#### 4.9.4. Gastrointestinal Histology

As in previous studies [14,15,16,18,20,21,22,23,24], we used a histologic scoring scale adapted from Chui and coworkers [98] for the stomach tissue damage, scoring 0–5 (normal to severe) in three categories (mucosal injury, inflammation, hyperemia/hemorrhage) for a total score of 0 to 15, as described by Lane and coworkers [99]. Illustratively, the assessment included morphologic features of mucosal injury (i.e., different grades of epithelial lifting, villi denudation, and necrosis), inflammation (i.e., focal to diffuse according to lamina propria infiltration or subendothelial infiltration), and hyperemia/hemorrhage (i.e., focal to diffuse according to lamina propria or subendothelial localization).

### 4.10. Statistical Analysis

Statistical analysis was performed by parametric one-way analysis of variance (ANOVA), with the Newman–Keuls post hoc test or the nonparametric Kruskal–Wallis test and, subsequently, the Mann–Whitney U test to compare groups. Values are presented as the mean ± standard deviation (SD) and as the minimum/median/maximum. To compare the frequency difference between groups, the chi-squared test or Fisher’s exact test was used. *p* < 0.05 was considered statistically significant.

## 5. Conclusions

In resolving the issue of the brain-gut and gut-brain axes (mal)functioning [1], we revealed as the new practical point, the central-peripheral and peripheral-central link, the occlusion/occlusion-like syndrome shared with dopamine agents. This implied haloperidol, fluphenazine, clozapine, olanzapine, risperidone, quetiapine, aripiprazole, domperidone, amphetamine, and combined amphetamine and haloperidol, and all particularities, consistently reversed by the stable gastric pentadecapeptide BPC 157. There, we start to highlight the significance of a full new topic (activation of collaterals, i.e., azygos vein as a common “bypassing key” for neuroleptic, domperidone, and amphetamine disturbances). Likewise, we initiate the essential matching of these findings with relationships that they are supposed to mimic (i.e., catalepsy, schizophrenia symptoms [28,29]). There is also the matching with the occlusion/occlusion-like syndromes induced by the major vessel(s) occlusion and other similar procedure applications [14,15,16,18,20,21,22,23,24]. Noteworthy, these congruent findings’ full significance remains to be further fully determined. However, these might be all consistently seen as a network of evidence for the physiologic significance of the revealed BPC 157/vascular-system interplay (i.e., BPC 157 was found in situ hybridization and immunostaining studies in humans to be largely distributed in tissues [8,91] and may have additional physiologic regulatory roles [1,2,3,4,5,6,7,8,9,10]). This issue was also approached by other reviews [100,101,102]. Based on the alike beneficial effects, similar importance was suggested also for other species (i.e., birds [103] and insects [104,105]). Moreover, there is also a very safe BPC 157 profile (i.e., no adverse effects in clinical trials (ulcerative colitis, phase II), and in toxicological studies, lethal dose (LD1) could be not achieved) (for review see [1,2,3,4,5,6,7,8,9,10,50,51,76,77,91]). This point was recently confirmed in a large study conducted by Xu and collaborators [106].

Together, these findings may be suggestive of the further BPC 157 therapy application.

## Figures and Tables

**Table 1:** Blood pressure (intracranial (superior sagittal sinus), portal and caval hypertension, and aortal hypotension, mm Hg) and thrombosis (thrombus mass, g) in rats at 15 min following intraperitoneal application of haloperidol (5 mg/kg) (H), fluphenazine (5 mg/kg) (F), clozapine (10 mg/kg) (C), risperidone (5 mg/kg) (R), olanzapine (10 mg/kg) (O), quetiapine (10 mg/kg) (Q), aripiprazole (10 mg/kg) (AR), domperidone (25 mg/kg) (D), amphetamine (10 mg/kg) (AM), and amphetamine (5 mg/kg) and haloperidol (5 mg/kg) (AM + H). Saline (5 mL/kg) (control) or BPC 157 (10 μg/kg and 10 ng/kg) were given ip at 5 min thereafter. * p ˂ 0.05, at least vs. *control*.

*Medication (ip) at 5 min after Dopamine Drug Application*	Blood Pressure and Thrombosis in Rats at 15 min Following Application of Haloperidol (H), Fluphenazine (F), Clozapine (C), Risperidone (R), Olanzapine (O), Quetiapine (Q), Aripiprazole (AR), Domperidone (D), Amphetamine (AM), and Amphetamine and Haloperidol (AM + H)									
	H	F	C	R	O	Q	AR	D	AM	AM + H
	Superior sagittal sinus pressure, mm Hg, Means ± SD									
Control	9 ± 1	8 ± 1	10 ± 2	12 ± 2	15 ± 2	13 ± 1	10 ± 1	5 ± 1	7 ± 1	8 ± 1
BPC 157 10 μg/kg	−3 ± 1 *	−1 ± 1 *	−1 ± 1 *	−1 ± 1 *	−7 ± 1 *	−2 ± 1 *	−1 ± 1 *	−1 ± 1 *	−1 ± 1 *	−1 ± 1 *
BPC 157 10 ng/kg	−5 ± 1 *	−2 ± 1 *	−1 ± 1 *	−1 ± 1 *	−6 ± 1 *	−2 ± 1 *	−1 ± 1 *	−1 ± 1 *	−1 ± 1 *	−1 ± 1 *
	**Portal pressure, mm Hg, Means ± SD**									
Control	17 ± 2	12 ± 1	15 ± 2	12 ± 1	18 ± 2	13 ± 2	12 ± 1	13 ± 1	13 ± 1	12 ± 1
BPC 157 10 μg/kg	5 ± 1 *	4 ± 1 *	4 ± 1 *	5 ± 1 *	4 ± 1 *	3 ± 1 *	4 ± 1 *	3 ± 1 *	4 ± 1 *	5 ± 1 *
BPC 157 10 ng/kg	5 ± 1 *	3 ± 1 *	2 ± 1 *	5 ± 1 *	5 ± 1 *	4 ± 1 *	5 ± 1 *	3 ± 1 *	4 ± 1 *	4 ± 1 *
	**Caval pressure, mm Hg, Means ± SD**									
Control	12 ± 2	11 ± 1	10 ± 1	11 ± 1	12 ± 2	10 ± 1	11 ± 1	11 ± 1	10 ± 2	10 ± 1
BPC 157 10 μg/kg	4 ± 1 *	3 ± 1 *	2 ± 1 *	4 ± 1 *	3 ± 1 *	3 ± 1 *	3 ± 1 *	2 ± 1 *	3 ± 1 *	4 ± 1 *
BPC 157 10 ng/kg	4 ± 1 *	3 ± 1 *	2 ± 1 *	4 ± 1 *	3 ± 1 *	3 ± 1 *	4 ± 1 *	2 ± 1 *	3 ± 1 *	4 ± 1 *
	**Aortal pressure, mm Hg, Means ± SD**									
Control	72 ± 5	55 ± 7	55 ± 8	53 ± 6	75 ± 5	52 ± 5	66 ± 5	52 ± 6	60 ± 5	55 ± 5
BPC 157 10 μg/kg	86 ± 4*	80 ± 5 *	73 ± 5 *	82 ± 5 *	90 ± 6 *	73 ± 5 *	78 ± 6 *	76 ± 5 *	75 ± 7 *	78 ± 7 *
BPC 157 10 ng/kg	90 ± 5 *	83 ± 7 *	75 ± 6 *	85 ± 8 *	92 ± 7 *	76 ± 6 *	79 ± 5 *	79 ± 7 *	77 ± 6 *	80 ± 5 *
	**Superior sagittal sinus, thrombus mass, g, Means ± SD**									
Control	0.0121 ± 0.0010	0.0062 ± 0.0009	0.081 ± 0.009	0.0047 ± 0.0006	0.049 ± 0.0008	0.086 ± 0.009	0.092 ± 0.01	0.0030 ± 0.0005	0.0028 ± 0.0005	0.0111 ± 0.005
BPC 157 10 μg/kg	*0.0031 ±* 0.0008 *	*0.0010 ±* 0.0007 *	*0.012 ±* 0.004 *	*0.0009 ±* 0.0004 *	*0.006 ±* 0.001 *	*0.015 ±* 0.004 *	*0.032 ±* 0.009 *	*0.0010 ±* 0.0006 *	*0.0007 ±* 0.0002 *	*0.0021 ±* 0.0005 *
BPC 157 10 ng/kg	*0.0035 ±* 0.0009 *	*0.0011 ±* 0.0005 *	*0.010 ±* 0.003 *	*0.0007 ±* 0.0003 *	*0.008 ±* 0.001 *	*0.012 ±* 0.004 *	*0.030 ±* 0.008 *	*0.0008 ±* 0.0003 *	*0.0005 ±* 0.0003 *	*0.0030 ±* 0.0005 *
	**Portal vein, thrombus mass, g, Means ± SD**									
Control	0.0265 ± 0.005	0.0106 ± 0.005	0.0127 ± 0.005	0.074 ± 0.008	0.079 ± 0.009	0.0107 ± 0.008	0.0107 ± 0.009	0.0046 ± 0.0009	0.0051 ± 0.0009	0.0165 ± 0.009
BPC 157 10 μg/kg	*0.003 ±* 0.0005 *	*0.0020 ±* 0.0005 *	*0.0015 ±* 0.0005 *	*0.032 ±* *0.008* *	*0.031 ±* 0.008 *	*0.0025 ±* 0.0009 *	*0.0035 ±* 0.0007 *	*0.0017 ±* 0.0005 *	*0.0021 ±* 0.0007 *	*0.0010 ±* 0.0009 *
BPC 157 10 ng/kg	*0.002 ±* 0.0007 *	*0.0018 ±* 0.0006 *	*0.0010 ±* 0.0005 *	*0.028 ±* 0.006 *	*0.033 ±* 0.008 *	*0.0020 ±* 0.0007 *	*0.0032 ±* 0.0009 *	*0.0015 ±* 0.0009 *	*0.0025 ±* 0.0006 *	*0.0015 ±* 0.0009 *
	**Inferior caval vein, thrombus mass, g, Means ± SD**									
Control	0.0238 ± 0.007	0.0171 ± 0.008	0.0123 ± 0.006	0.0091 ± 0.0007 *	0.0089 ± 0.0009	0.0095 ± 0.0008	0.0103 ± 0.005	0.0051 ± 0.0009	0.0040 ± 0.0007 *	0.0138 ± 0.007
BPC 157 10 μg/kg	*0.0072 ±* 0.0007 *	*0.0071 ±* 0.0008 *	*0.0050 ±* 0.0009 *	*0.0041 ±* 0.0008 *	*0.0035 ±* 0.0007 *	*0.0047 ±* 0.0008 *	*0.0040 ±* 0.0007 *	*0.0020 ±* 0.0007 *	*0.0018 ±* 0.0007 *	*0.0052 ±* 0.0009 *
BPC 157 10 ng/kg	*0.0068 ±* 0.0008 *	*0.0065 ±* 0.0008 *	*0.0055 ±* 0.0007 *	*0.0035 ±* 0.0006 *	*0.0038 ±* 0.0007 *	*0.0045 ±* 0.0007 *	*0.0045 ±* 0.0009 *	*0.0016 ±* 0.0006 *	*0.0020 ±* 0.0008 *	*0.0048 ±* 0.0007 *
	**Abdominal aorta, thrombus mass, g, Means ± SD**									
Control	0.0204 ± 0.007	0.0113 ± 0.009	0.0101 ± 0.007	0.0081 ± 0.0009	0.0070 ± 0.001	0.0101 ± 0.007	0.0091 ± 0.001	0.0055 ± 0.0009	0.0047 ± 0.007	0.0104 ± 0.007
BPC 157 10 μg/kg	*0.0036 ±* 0.0005 *	*0.0060 ±* 0.0007 *	*0.0042 ±* 0.0005 *	*0.0041 ±* 0.0008 *	*0.0029 ±* 0.0005 *	*0.0045 ±* 0.0006 *	*0.0032 ±* 0.0007 *	*0.0025 ±* 0.0006 *	*0.0021 ±* 0.0007 *	*0.0026 ±* 0.0008 *
BPC 157 10 ng/kg	*0.0040 ±* 0.0007 *	*0.0055 ±* 0.0009 *	*0.0035 ±* 0.0007 *	*0.0043 ±* 0.0007 *	*0.0025 ±* 0.0005 *	*0.0048 ±* 0.0007 *	*0.0038 ±* 0.0008 *	*0.0022 ±* 0.0007 *	*0.0018 ±* 0.0008 *	*0.0030 ±* 0.0009 *

**Table 2:** Relative volume (control/treated, %) of the brain, heart, azygos vein, inferior caval vein, superior mesenteric vein, and abdominal aorta in rats at 15 min following intraperitoneal application of haloperidol (5 mg/kg) (H), fluphenazine (5 mg/kg) (F), clozapine (10 mg/kg) (C), risperidone (5 mg/kg) (R), olanzapine (10 mg/kg) (O), quetiapine (10 mg/kg) (Q), aripiprazole (10 mg/kg) (AR), domperidone (25 mg/kg) (D), amphetamine (10 mg/kg) (AM), and amphetamine (5 mg/kg) combined with haloperidol (5 mg/kg) (AM + H). Saline (5 mL/kg) (control) or BPC 157 (10 μg/kg and 10 ng/kg) were given ip at 5 min thereafter. * p ˂ 0.05, at least vs. *control*.

*Medication (ip) at 5 min after Dopamine Drug Application*	Relative Volume (Control/Treated) (%) of the Brain, Heart, Azygos Vein, Inferior Caval Vein, Superior Mesenteric Vein, and Abdominal Aorta in Rats at 15 min Following Application of Haloperidol (H), Fluphenazine (F), Clozapine (C), Risperidone (R), Olanzapine (O), Quetiapine (Q), Aripiprazole (AR), Domperidone (D), Amphetamine (AM), and Amphetamine and Haloperidol (AM + H)									
	H	F	C	R	O	Q	AR	D	AM	AM + H
	Relative volume (control/treated) (%) of the brain, Means ± SD									
BPC 157 10 μg/kg	113 ± 3 *	118 ± 2 *	109 ± 3 *	115 ± 3 *	109 ± 3 *	111 ± 3 *	117 ± 2 *	108 ± 2 *	112 ± 3 *	115 ± 3 *
BPC 157 10 ng/kg	111 ± 3 *	115 ± 3 *	111 ± 2 *	116 ± 2 *	110 ± 2 *	113 ± 2 *	115 ± 3 *	110 ± 2 *	110 ± 3 *	117 ± 3 *
	**Relative volume (control/treated) (%) of the heart, Means ± SD**									
BPC 157 10 μg/kg	117 ± 4 *	121 ± 5 *	110 ± 3 *	108 ± 3 *	147 ± 6 *	138 ± 6 *	120 ± 4 *	123 ± 3 *	111 ± 3 *	126 ± 5 *
BPC 157 10 ng/kg	119 ± 3 *	124 ± 4 *	113 ± 4 *	110 ± 3 *	144 ± 7 *	135 ± 7 *	125 ± 5 *	120 ± 5 *	114 ± 3 *	129 ± 6 *
	**Relative volume (control/treated) (%) of the azygos vein, Means ± SD**									
BPC 157 10 μg/kg	69 ± 6 *	40 ± 6 *	31 ± 6 *	43 ± 4 *	36 ± 6 *	50 ± 6 *	43 ± 6 *	25 ± 6 *	45 ± 7 *	39 ± 6 *
BPC 157 10 ng/kg	66 ± 4 *	44 ± 7 *	29 ± 5 *	*40 ± 6*	33 ± 5 *	52 ± 7 *	45 ± 5 *	27 ± 7 *	47 ± 6 *	41 ± 7 *
	**Relative volume (control/treated) (%) of the inferior caval vein, Means ± SD**									
BPC 157 10 μg/kg	151 ± 10 *	139 ± 6 *	151 ± 9 *	*112 ± 5*	*206 ± 12*	127 ± 6 *	123 ± 8 *	125 ± 6 *	*117 ± 5*	134 ± 9 *
BPC 157 10 ng/kg	155 ± 12 *	136 ± 7 *	155 ± 12 *	116 ± 6 *	*202 ± 14*	125 ± 7 *	120 ± 9 *	128 ± 8 *	119 ± 7 *	136 ± 8 *
	**Relative volume (control/treated) (%) of the superior mesenteric vein, Means ± SD**									
BPC 157 10 μg/kg	189 ± 12 *	269 ± 22 *	260 ± 15 *	218 ± 14 *	233 ± 19 *	167 ± 17 *	137 ± 12 *	132 ± 15 *	268 ± 18 *	184 ± 16 *
BPC 157 10 ng/kg	199 ± 19 *	259 ± 24 *	270 ± 14 *	226 ± 19 *	239 ± 16 *	158 ± 15 *	144 ± 16 *	138 ± 17 *	255 ± 22 *	188 ± 18 *
	**Relative volume (control/treated) (%) of the abdominal aorta, Means ± SD**									
BPC 157 10 μg/kg	69 ± 10 *	66 ± 12 *	58 ± 14 *	58 ± 9 *	67 ± 8 *	77 ± 9 *	55 ± 11 *	60 ± 10 *	70 ± 8 *	78 ± 7 *
BPC 157 10 ng/kg	66 ± 7 *	68 ± 10 *	62 ± 12 *	62 ± 8 *	64 ± 7 *	73 ± 6 *	50 ± 9 *	58 ± 8 *	66 ± 7 *	74 ± 9 *

**Table 3:** ECG changes (PQ intervals and QTc intervals, msec; heart frequency, beats/min) in rats at 15 min following intraperitoneal application of haloperidol (5 mg/kg) (H), fluphenazine (5 mg/kg) (F), clozapine (10 mg/kg) (C), risperidone (5 mg/kg) (R), olanzapine (10 mg/kg) (O), quetiapine (10 mg/kg) (Q), aripiprazole (10 mg/kg) (AR), domperidone (25 mg/kg) (D), amphetamine (10 mg/kg) (AM), and amphetamine (5 mg/kg) and haloperidol (5 mg/kg) (AM + H). Saline (5 mL/kg) (control) or BPC 157 (10 μg/kg and 10 ng/kg) were given ip at 5 min thereafter. * p ˂ 0.05, at least vs. *control*.

*Medication (ip) at 5 min after Dopamine Drug Application*	ECG Changes in Rats at 15 min Following Application of Haloperidol (H), Fluphenazine (F), Clozapine (C), Risperidone (R), Olanzapine (O), Quetiapine (Q), Aripiprazole (AR), Domperidone (D), Amphetamine (AM), and Amphetamine and Haloperidol (AM + H)									
	H	F	C	R	O	Q	AR	D	AM	AM + H
	PQ interval, msec Means ± SD									
Control	65 ± 5	66 ± 4	67 ± 4	65 ± 5	68 ± 5	65 ± 5	66 ± 5	65 ± 5	65 ± 5	50 ± 5
BPC 157 10 μg/kg	50 ± 5 *	50 ± 5 *	50 ± 5 *	50 ± 5 *	50 ± 5 *	50 ± 5 *	50 ± 5 *	50 ± 5 *	50 ± 5 *	50 ± 5
BPC 157 10 ng/kg	50 ± 5 *	50 ± 5 *	50 ± 5 *	50 ± 5 *	50 ± 5 *	50 ± 5 *	50 ± 5 *	50 ± 5 *	50 ± 5 *	50 ± 5
	**QTc interval, msec, Means ± SD**									
Control	241 ± 10	247 ± 10	251 ± 10	255 ± 10	248 ± 10	254 ± 10	250 ± 10	251 ± 10	175 ± 5	190 ± 10
BPC 157 10 μg/kg	187 ± 10 *	185 ± 10 *	190 ± 10 *	185 ± 10 *	185 ± 10 *	188 ± 10 *	185 ± 10 *	195 ± 10 *	195 ± 5 *	185 ± 10
BPC 157 10 ng/kg	186 ± 10 *	190 ± 10 *	190 ± 10 *	190 ± 10 *	187 ± 10 *	190 ±10 *	190 ± 10 *	190 ± 10 *	190 ± 5 *	190 ± 10
	**Heart frequency, beats/min, Means ± SD**									
Control	430 ± 10	450 ± 10	465 ± 10	485 ± 10	480 ± 10	445 ± 10	485 ± 10	465 ± 10	440 ± 10	440 ± 10
BPC 157 10 μg/kg	370 ± 10 *	350 ± 10 *	380 ± 10 *	360 ± 10 *	375 ± 10 *	355 ± 10 *	390 ± 10 *	370 ± 10 *	405 ± 10 *	395 ± 10 *
BPC 157 10 ng/kg	365 ± 10 *	355 ± 10 *	375 ± 10 *	355 ± 10 *	385 ± 10 *	350 ± 10 *	385 ± 10 *	375 ± 10 *	400 ± 10 *	390 ± 10 *

**Table 4:** Lesions scored microscopically (heart, lung, liver, kidney, stomach) or macroscopically (stomach) in rats at 15 min following intraperitoneal application of haloperidol (5 mg/kg) (H), fluphenazine (5 mg/kg) (F), clozapine (10 mg/kg) (C), risperidone (5 mg/kg) (R), olanzapine (10 mg/kg) (O), quetiapine (10 mg/kg) (Q), aripiprazole (10 mg/kg) (AR), domperidone (25 mg/kg) (D), amphetamine (10 mg/kg) (AM), and amphetamine (5 mg/kg) and haloperidol (5 mg/kg) (AM + H). Saline (5 mL/kg) (control) or BPC 157 (10 μg/kg and 10 ng/kg) were given ip at 5 min thereafter. * p ˂ 0.05, at least vs. *control*.

*Medication (ip) at 5 min after Dopamine Drug Application*	Lesions Scored Microscopically (Heart, Lung, Liver, Kidney, and Stomach) or Macroscopically (Stomach) in Rats at 15 min Following Application of Haloperidol (H), Fluphenazine (F), Clozapine (C), Risperidone (R), Olanzapine (O), Quetiapine (Q), Aripiprazole (AR), Domperidone (D), Amphetamine (AM), and Amphetamine and Haloperidol (AM + H)									
	H	F	C	R	O	Q	AR	D	AM	AM + H
	Heart (scored 0–3, Min/Med/Max)									
Control	3/3/3	2/3/3	2/3/3	2/2/3	2/2/2	2/3/3	2/2/2	2/3/3	2/2/2	2/2/2
BPC 157 10 μg/kg	1/1/1 *	0/0/0 *	1/1/1 *	0/1/1 *	0/1/1 *	1/2/2 *	1/1/1 *	1/1/1 *	0/0/0 *	0/0/0 *
BPC 157 10 ng/kg	1/1/1 *	0/0/0 *	1/1/1 *	0/1/1 *	0/1/1 *	1/2/2 *	1/1/1 *	1/1/1 *	0/0/0 *	0/0/0 *
	**Lung (scored 0–3, Min/Med/Max)**									
Control	2/2/2	1/2/2	3/3/3	3/3/3	1/2/2	2/2/3	1/2/2	2/2/2	2/3/3	2/3/3
BPC 157 10 μg/kg	1/1/2 *	0/0/0 *	1/1/1 *	0/1/1 *	1/1/1 *	1/1/2 *	0/1/1 *	0/1/1 *	0/1/1 *	0/0/0 *
BPC 157 10 ng/kg	1/1/2 *	0/0/0 *	1/1/1 *	0/1/1 *	1/1/1 *	1/1/2 *	0/1/1 *	0/1/1 *	0/1/1 *	0/0/0 *
	**Liver (scored 0–3, Min/Med/Max)**									
Control	2/2/2	2/3/3	2/3/3	2/3/3	2/3/3	2/3/3	2/3/3	2/2/2	2/2/2	3/3/3
BPC 157 10 μg/kg	0/0/0 *	0/1/1 *	0/1/1 *	0/1/1 *	1/1/1 *	0/1/1 *	0/0/0 *	0/1/1 *	0/0/0 *	0/0/0 *
BPC 157 10 ng/kg	0/0/0 *	0/1/1 *	0/1/1 *	0/1/1 *	1/1/1 *	0/1/1 *	0/0/0 *	0/1/1 *	0/0/0 *	0/0/0 *
	**Kidney (scored 0–3, Min/Med/Max)**									
Control	2/2/2	2/2/2	2/2/2	1/2/2	2/2/2	2/3/3	2/3/3	2/3/3	3/3/3	2/2/2
BPC 157 10 μg/kg	0/1/1 *	0/1/1 *	0/1/1 *	0/1/1 *	0/1/1 *	1/1/1 *	1/1/1 *	1/1/1 *	1/1/1 *	0/1/1 *
BPC 157 10 ng/kg	0/1/1 *	0/1/1 *	0/1/1 *	0/1/1 *	1/1/1 *	0/1/1 *	1/1/1 *	0/1/1 *	1/1/1 *	0/1/1 *
	**Stomach (sum of longest diameters, mm, Means ± SD)**									
Control	11 ± 2	5 ± 1	5 ± 2	5 ± 1	10 ± 2	3 ± 1	3 ± 1	12 ± 3	10 ± 2	10 ± 3
BPC 157 10 μg/kg	0 ± 0 *	0 ± 0 *	0 ± 0 *	0 ± 0 *	0 ± 0 *	0 ± 0 *	0 ± 0 *	0 ± 0 *	0 ± 0 *	0 ± 0 *
BPC 157 10 ng/kg	0 ± 0 *	0 ± 0 *	0 ± 0 *	0 ± 0 *	0 ± 0 *	0 ± 0 *	0 ± 0 *	0 ± 0 *	0 ± 0 *	0 ± 0 *
	**Stomach (scored 0–15, Min/Med/Max)**									
Control	2/2/2	1/1/1	1/1/1	2/2/2	2/2/2	2/2/2	1/2/2	1/2/2	1/1/1	2/3/3
BPC 157 10 μg/kg	0/0/0 *	0/0/0 *	0/0/0 *	0/0/0 *	0/0/0 *	0/0/0 *	0/0/0 *	0/0/0 *	0/0/0 *	0/0/0 *
BPC 157 10 ng/kg	0/0/0 *	0/0/0 *	0/0/0 *	0/0/0 *	0/0/0 *	0/0/0 *	0/0/0 *	0/0/0 *	0/0/0 *	0/0/0 *

**Table 5:** Lesions were assessed microscopically (cerebrum, cerebellum, hypothalamus, hippocampus) in rats at 15 min following intraperitoneal application of haloperidol (5 mg/kg) (H), fluphenazine (5 mg/kg) (F), clozapine (10 mg/kg) (C), risperidone (5 mg/kg) (R), olanzapine (10 mg/kg) (O), quetiapine (10 mg/kg) (Q), aripiprazole (10 mg/kg) (AR), domperidone (25 mg/kg) (D), amphetamine (10 mg/kg) (AM), and amphetamine (5 mg/kg) combined with haloperidol (5 mg/kg) (AM + H). Saline (5 mL/kg) (control) or BPC 157 (10 μg/kg and 10 ng/kg) were given ip at 5 min thereafter. * p ˂ 0.05, at least vs. *control*.

*Medication (ip) at 5 min after Dopamine Drug Application*	Lesions Scored Microscopically in the Cerebrum, Cerebellum, Hypothalamus, and Hippocampus in Rats at 15 min Following Application of Haloperidol (H), Fluphenazine (F), Clozapine (C), Risperidone (R), Olanzapine (O), Quetiapine (Q), Aripiprazole (AR), Domperidone (D), Amphetamine (AM), and Amphetamine and Haloperidol (AM + H)									
	H	F	C	R	O	Q	AR	D	AM	AM + H
	Cerebrum (scored 0–8, Min/Med/Ma) Percentage of karyopyknotic cells (%), Means ± SD									
Control	2/2/2 40 ± 5	½/2 35 ± 5	1/1/1 20 ± 5	1/1/2 25 ± 5	2/2/2 30 ± 5	2/2/2 30 ± 5	2/3/3 55 ± 5	2/3/3 60 ± 5	2/3/3 65 ± 5	3/3/3 60 ± 5
BPC 157 10 μg/kg	0/0/1 * 10 ± 10 *	*1/1/1* 10 ± 5 *	0/0/1 * 5 ± 5 *	0/0/1 * 5 ± 5 *	0/0/1 * 5 ± 5 *	0/0/1 * 5 ± 5 *	1/1/2 * 20 ± 10 *	1/1/2 * 20 ± 10 *	1/1/2 * 20 ± 10 *	0/0/1 * 10 ± 10 *
BPC 157 10 ng/kg	0/0/1 * 10 ± 10 *	*1/1/1* 10 ± 5 *	0/0/1 * 5 ± 5 *	0/0/1 * 5 ± 5 *	0/0/1 * 5 ± 5 *	0/0/1 * 5 ± 5 *	1/1/2 * 20 ± 10 *	1/1/2 * 20 ± 10 *	1/1/2 * 20 ± 10 *	0/0/1 * 10 ± 10 *
	**Neuronal damage in the karyopyknotic areas, %, Means ± SD (10 HPF, 400×)**									
Control	10 ± 2	10 ± 1	17 ± 3	15 ± 2	23 ± 3	10 ± 3	15 ± 3	32 ± 4	24 ± 3	33 ± 3
BPC 157 10 μg/kg	2 ± 2 *	3 ± 2 *	9 ± 2 *	7 ± 2 *	3 ± 3 *	4 ± 2 *	*10 ± 1*	13 ± 2 *	7 ± 2 *	19 ± 2 *
BPC 157 10 ng/kg	2 ± 2 *	2 ± 2 *	10 ± 2 *	5 ± 2 *	3 ± 3 *	4 ± 2 *	*9 ± 1*	12 ± 2 *	6 ± 2 *	18 ± 2 *
	**Hemorrhage (% of total area), Means ± SD**									
Control	40 ± 5	20 ± 2	0 ± 0	0 ± 0	40 ± 5	20 ± 2	20 ± 3	40 ± 5	40 ± 5	60 ± 5
BPC 157 10 μg/kg	10 ± 3 *	10 ± 5 *	0 ± 0	0 ± 0	10 ± 5 *	10 ± 3 *	10 ± 3 *	20 ± 3 *	10 ± 3 *	20 ± 5 *
BPC 157 10 ng/kg	9 ± 3 *	8 ± 4 *	0 ± 0	0 ± 0	9 ± 3 *	9 ± 4 *	11 ± 3 *	20 ± 3 *	10 ± 3 *	20 ± 5 *
	**Edema (scored 0–3, Min/Med/Max)**									
Control	3/3/3	3/3/3	2/3/3	3/3/3	3/3/3	2/3/3	2/3/3	2/3/3	2/3/3	3/3/3
BPC 157 10 μg/kg	1/1/1 *	1/1/1 *	0/1/1 *	1/1/2 *	1/1/1 *	1/1/1 *	1/1/2 *	1/1/2 *	2/2/2 *	2/2/2 *
BPC 157 10 ng/kg	1/1/1 *	1/1/1 *	0/1/1 *	1/1/2 *	1/1/1 *	1/1/1 *	1/1/2 *	1/1/2 *	2/2/2 *	2/2/2 *
	**Cerebellum (scored 0–8, Min/Med/Ma)** **Percentage of karyopyknotic cells (%), Means ± SD**									
Control	½/2 30 ± 10	1/1/1 20 ± 2	1/1/1 20 ± 2	1/1/1 18 ± 2	1/1/1 20 ± 2	½/2 30 ± 2	2/3/3 55 ± 5	1/1/1 20 ± 2	1/1/1 20 ± 2	2/3/3 60 ± 5
BPC 157 10 μg/kg	0/1/1 * 5 ± 5 *	0/1/1 * 3 ± 2 *	1/1/1 * 3 ± 2 *	0/1/1 * 3 ± 2 *	0/1/1 * 3 ± 2 *	1/1/1 * 7 ± 2 *	1/1/1 * 16 ± 4 *	0/1/1 * 3 ± 2 *	1/1/1 * 7 ± 2 *	1/1/1 * 7 ± 2 *
BPC 157 10 ng/kg	0/1/1 * 5 ± 5 *	0/1/1 * 3 ± 2 *	1/1/1 * 3 ± 2 *	0/1/1 * 3 ± 2 *	0/1/1 * 3 ± 2 *	1/1/1 * 7 ± 2 *	1/1/1 * 15 ± 5 *	0/1/1 * 3 ± 2 *	1/1/1 * 6 ± 2 *	1/1/1 * 6 ± 2 *
	**Neuronal damage in the karyopyknotic areas, %, Means ± SD (10 HPF, 400×)**									
Control	3 ± 1	7 ± 2	8 ± 3	10 ± 2	4 ± 1	8 ± 1	15 ± 2	5 ± 1	5 ± 2	18 ± 1
BPC 157 10 μg/kg	0 ± 0 *	3 ± 1 *	1 ± 1 *	1 ± 1 *	1 ± 1 *	3 ± 1 *	4 ± 1 *	1 ± 1 *	1 ± 1 *	*4 ± 2*
BPC 157 10 ng/kg	0 ± 0 *	3 ± 1 *	1 ± 1 *	1 ± 1 *	1 ± 1 *	2 ± 1 *	3 ± 1 *	1 ± 1 *	1 ± 1 *	3 ± 1 *
	**Hemorrhage (% of total area), Means ± SD**									
Control	0 ± 0	0 ± 0	0 ± 0	0 ± 0	0 ± 0	0 ± 0	0 ± 0	0 ± 0	0 ± 0	0 ± 0
BPC 157 10 μg/kg	0 ± 0	0 ± 0	0 ± 0	0 ± 0	0 ± 0	0 ± 0	0 ± 0	0 ± 0	0 ± 0	0 ± 0
BPC 157 10 ng/kg	0 ± 0	0 ± 0	0 ± 0	0 ± 0	0 ± 0	0 ± 0	0 ± 0	0 ± 0	0 ± 0	0 ± 0
	**Edema (scored 0–3, Min/Med/Max)**									
Control	3/3/3	2/2/2	2/3/3	2/2/2	2/3/3	2/2/2	2/3/3	2/3/3	2/2/2	2/3/3
BPC 157 10 μg/kg	1/1/1 *	1/1/1 *	0/1/1 *	0/1/1 *	0/1/1 *	1/1/1 *	0/1/1 *	0/1/1 *	1/1/1 *	1/1/1 *
BPC 157 10 ng/kg	1/1/1 *	1/1/1 *	0/1/1 *	0/1/1 *	0/1/1 *	1/1/1 *	0/1/1 *	0/1/1 *	1/1/1 *	1/1/1 *
	**Hippocampus (scored 0–8, Min/Med/Max)** **Percentage of karyopyknotic cells (%), Means ± SD**									
Control	1/1/1 13 ± 3	1/1/1 10 ± 1	1/1/1 10 ± 1	1/1/1 5 ± 0	1/1/1 10 ± 1	1/1/1 10 ± 1	1/1/1 10 ± 1	1/1/1 10 ± 1	1/1/1 10 ± 1	1/1/1 10 ± 1
BPC 157 10 μg/kg	0/1/1 * 3 ± 1 *	0/1/1 * 3 ± 1 *	0/1/1 * 3 ± 1 *	0/1/1 * 3 ± 1 *	0/0/0 * 0 ± 0 *	0/1/1 * 3 ± 1 *	0/1/1 * 2 ± 1 *	0/1/1 * 3 ± 1 *	0/1/1 * 3 ± 1 *	0/1/1 * 3 ± 1 *
BPC 157 10 ng/kg	0/1/1 * 3 ± 1 *	0/1/1 * 3 ± 1 *	0/1/1 * 3 ± 1 *	0/1/1 * 3 ± 1 *	0/0/0 * 0 ± 0 *	0/1/1 * 3 ± 1 *	0/1/1 * 2 ± 1 *	0/1/1 * 3 ± 1 *	0/1/1 * 3 ± 1 *	0/1/1 * 3 ± 1 *
	**Neuronal damage in the karyopyknotic areas, %, Means ± SD (10 HPF, 400×)**									
Control	3 ± 1	7 ± 1	2 ± 1	2 ± 1	9 ± 1	3 ± 1	6 ± 1	7 ± 1	2 ± 1	4 ± 1
BPC 157 10 μg/kg	1 ± 0 *	1 ± 1 *	1 ± 0 *	1 ± 0 *	0 ± 0 *	1 ± 0 *	1 ± 0 *	1 ± 0 *	1 ± 0 *	2 ± 1 *
BPC 157 10 ng/kg	1 ± 0 *	1 ± 1 *	1 ± 0 *	1 ± 0 *	0 ± 0 *	1 ± 0 *	1 ± 0 *	1 ± 0 *	1 ± 0 *	2 ± 1 *
	**Hemorrhage (% of total area), Means ± SD**									
Control	0 ± 0	0 ± 0	0 ± 0	0 ± 0	0 ± 0	0 ± 0	0 ± 0	0 ± 0	0 ± 0	0 ± 0
BPC 157 10 μg/kg	0 ± 0	0 ± 0	0 ± 0	0 ± 0	0 ± 0	0 ± 0	0 ± 0	0 ± 0	0 ± 0	0 ± 0
BPC 157 10 ng/kg	0 ± 0	0 ± 0	0 ± 0	0 ± 0	0 ± 0	0 ± 0	0 ± 0	0 ± 0	0 ± 0	0 ± 0
	**Edema (scored 0–3, Min/Med/Max)**									
Control	2/2/2	2/3/3	2/3/3	2/3/3	2/3/3	½/3	2/2/2	2/2/2	2/2/2	2/2/2
BPC 157 10 μg/kg	0/1/1 *	0/1/1 *	0/1/1 *	0/1/1 *	0/0/0 *	0/1/1 *	0/0/0 *	0/1/1 *	0/1/1 *	1/1/1 *
BPC 157 10 ng/kg	0/1/1 *	0/1/1 *	0/1/1 *	0/1/1 *	0/0/0 *	0/1/1 *	0/0/0 *	0/1/1 *	0/1/1 *	1/1/1 *
	**Hypothalamus (scored 0–8, Min/Med/Max)** **Percentage of karyopyknotic cells (%), Means ± SD**									
Control	1/1/1 13 ± 2	1/1/1 13 ± 2	1/1/1 5 ± 1	1/1/1 5 ± 1	2/2/2 37 ± 3	1/1/1 20 ± 2	2/2/2 40 ± 2	1/1/1 10 ± 1	2/2/2 40 ± 1	2/2/2 30 ± 2
BPC 157 10 μg/kg	0/1/1 * 3 ± 2 *	0/1/1 * 3 ± 2 *	0/1/1 * 3 ± 2 *	0/1/1 * 3 ± 2 *	0/1/1 * 3 ± 2 *	0/1/1 * 3 ± 2 *	1/1/1 * 20 ± 2 *	1/1/1 * 5 ± 2 *	1/1/1 * 10 ± 1 *	1/1/1 * *16 ± 4*
BPC 157 10 ng/kg	0/1/1 * 3 ± 2 *	0/1/1 * 3 ± 2 *	0/1/1 * 3 ± 2 *	0/1/1 * 3 ± 2 *	0/1/1 * 3 ± 2 *	0/1/1 * 3 ± 2 *	1/1/1 * 20 ± 2 *	1/1/1 * 5 ± 3 *	1/1/1 * 10 ± 1 *	1/1/1 * 15 ± 5 *
	**Neuronal damage in the karyopyknotic areas, %, Means ± SD (10 HPF, 400×)**									
Control	4 ± 1	8 ± 2	6 ± 2	6 ± 2	22 ± 1	7 ± 1	22 ± 6	7 ± 1	22 ± 2	19 ± 3
BPC 157 10 μg/kg	1 ± 1 *	1 ± 1 *	1 ± 1 *	2 ± 1 *	3 ± 2 *	1 ± 1 *	9 ± 3 *	3 ± 1 *	5 ± 2 *	9 ± 2 *
BPC 157 10 ng/kg	1 ± 1 *	1 ± 1 *	1 ± 1 *	2 ± 1 *	3 ± 2 *	1 ± 1 *	8 ± 3 *	3 ± 1 *	5 ± 3 *	10 ± 2 *
	**Hemorrhage (% of total area), Means ± SD**									
Control	0 ± 0	0 ± 0	0 ± 0	0 ± 0	0 ± 0	0 ± 0	0 ± 0	0 ± 0	0 ± 0	0 ± 0
BPC 157 10 μg/kg	0 ± 0	0 ± 0	0 ± 0	0 ± 0	0 ± 0	0 ± 0	0 ± 0	0 ± 0	0 ± 0	0 ± 0
BPC 157 10 ng/kg	0 ± 0	0 ± 0	0 ± 0	0 ± 0	0 ± 0	0 ± 0	0 ± 0	0 ± 0	0 ± 0	0 ± 0
	**Edema (scored 0–3, Min/Med/Max)**									
Control	3/3/3	2/2/2	2/2/2	2/2/2	2/3/3	2/2/2	2/3/3	2/2/2	2/3/3	2/3/3
BPC 157 10 μg/kg	0/1/1 *	0/1/1 *	0/1/1 *	0/1/1 *	0/1/1 *	0/1/1 *	0/1/1 *	0/1/1 *	1/1/1 *	2/2/2 *
BPC 157 10 ng/kg	0/1/1 *	0/1/1 *	0/1/1 *	0/1/1 *	0/1/1 *	0/1/1 *	0/1/1 *	0/1/1 *	1/1/1 *	2/2/2 *

## Data Availability

The data presented in this study are available on request from the corresponding authors.
